# Important contributions of non-fossil fuel nitrogen oxides emissions

**DOI:** 10.1038/s41467-020-20356-0

**Published:** 2021-01-11

**Authors:** Wei Song, Xue-Yan Liu, Chao-Chen Hu, Guan-Yi Chen, Xue-Jun Liu, Wendell W. Walters, Greg Michalski, Cong-Qiang Liu

**Affiliations:** 1 School of Earth System Science, Tianjin University, 300072 Tianjin, China; 2 Georgia Tech Shenzhen Institute, Tianjin University, 518071 Shenzhen, China; 3 College of Resources and Environmental Sciences, China Agricultural University, 100193 Beijing, China; 4 Institute at Brown for Environment and Society, Brown University, 85 Waterman St, Providence, RI 02912 USA; 5 Department of Earth, Atmospheric, and Planetary Sciences, Purdue University, 550 Stadium Mall Drive, West Lafayette, IN 47907 USA

**Keywords:** Element cycles, Stable isotope analysis, Geochemistry, Environmental impact

## Abstract

Since the industrial revolution, it has been assumed that fossil-fuel combustions dominate increasing nitrogen oxide (NO_x_) emissions. However, it remains uncertain to the actual contribution of the non-fossil fuel NO_x_ to total NO_x_ emissions. Natural N isotopes of NO_3_^−^ in precipitation (δ^15^N_w-NO3−_) have been widely employed for tracing atmospheric NO_x_ sources. Here, we compiled global δ^15^N_w-NO3−_ observations to evaluate the relative importance of fossil and non-fossil fuel NO_x_ emissions. We found that regional differences in human activities directly influenced spatial-temporal patterns of δ^15^N_w-NO3−_ variations. Further, isotope mass-balance and bottom-up calculations suggest that the non-fossil fuel NO_x_ accounts for 55 ± 7% of total NO_x_ emissions, reaching up to 21.6 ± 16.6Mt yr^−1^ in East Asia, 7.4 ± 5.5Mt yr^−1^ in Europe, and 21.8 ± 18.5Mt yr^−1^ in North America, respectively. These results reveal the importance of non-fossil fuel NO_x_ emissions and provide direct evidence for making strategies on mitigating atmospheric NO_x_ pollution.

## Abstract (web summary)

This study investigates in the importance of non-fossil fuel NO_x_ emissions in the surface-earth-nitrogen cycle. The study shows how changes of regional human activities directly influence δ^15^N signatures of deposited NO_x_ to terrestrial environments and that emissions have largely been underestimated.

## Introduction

Over past decades, both concentrations and deposition fluxes of nitrogen oxides (NO_*x*_), nitric acid (HNO_3_), and nitrate (NO_3_^−^) in the atmosphere have been remarkably elevated in many regions of the world^1–4^. This has caused negative effects on the environmental quality (e.g., haze, eutrophication), human health (e.g., respiratory and cardiovascular diseases, acute bronchitis), and the structure and functions of ecosystems (e.g., soil acidification, biodiversity losses)^5,6^. Gaseous NO_*x*_, the sum of N oxide (NO) and N dioxide (NO_2_), is the precursor of atmospherically deposited NO_3_^−^ ^7,8^ and mainly emitted from fossil fuel combustion (primarily via coal combustion and vehicle exhausts) and non-fossil fuel sources including biomass burning, microbial N cycles in soils and animal wastes^9^. Accurate differentiation of NO_*x*_ emissions from fossil-fuel and non-fossil emission sectors is pivotal for regulatory action to mitigate emissions, budget NO_3_^−^ deposition fluxes, and model ecological and climatic effects of atmospheric NO_3_^−^ loading.

It is feasible to estimate fossil fuel NO_*x*_ emissions according to known consumption amounts of fossil fuels and their NO_*x*_ emission factors^10–12^. More often, fossil fuel NO_*x*_ emissions in many countries have been recorded in national statistics yearbooks and emission inventories^2,12–15^. Since the 1990s, fossil fuel NO_*x*_ emissions have accounted for 95% of global NO_*x*_ emissions^11^, 90% of NO_*x*_ emissions in Europe^2^, 88% of NO_*x*_ emissions in East Asia^10^, and 96% of NO_*x*_ emissions in North America^14,15^. In contrast, the importance and amount of non-fossil fuel NO_*x*_ emissions remain unclear due to the difficulties in obtaining their emission factors and amounts. Particularly, it is almost impossible to budget NO_*x*_ emission amounts from diverse biomass burnings and microbial N cycles that occur in different solid- and liquid-phase substrates^16–18^. In many cases, data of emission factors and estimates of emission budgets were rather incomplete and even unrecorded for non-fossil fuel NO_*x*_.

However, according to the simulation results of atmospheric chemical transport and terrestrial ecosystem models, biomass burning and soil emissions account for about 20% and 22% of global NO_*x*_ emissions, respectively^19–21^. The combination of a bottom-up spatial model and top-down airborne observations of atmospheric NO_*x*_ concentrations through satellite imagery pointed to a significant and overlooked NO_*x*_ emission from cropland soils, which constitutes 20–51% of the total NO_*x*_ budget at the regional scale^22^. Recently, natural stable N isotopes (expressed as δ^15^N, δ^15^N = (^15^N*/*^14^N)_sample_/(^15^N*/*^14^N)_standard_ −1, where atmospheric N_2_ is used as the internationally recognized N isotopic standard) have been widely employed for tracking NO_*x*_ emissions^7,23–25^. Isotopic investigations have demonstrated that NO_*x*_ from biomass burning and microbial N cycle may account for more than 40% of NO_3_^−^ in particulates and precipitation collected in urban sites of China^26,27^. In summary, we argue that the importance of non-fossil fuel NO_*x*_ is still an open question.

NO is the most initial form of fossil fuel and non-fossil fuel NO_*x*_ emissions, but NO is normally insoluble and will be rapidly oxidized to NO_2_ in the atmosphere, forming the photochemical NO_*x*_ cycle^28^. The mixing of fossil fuel and non-fossil fuel NO_*x*_ emissions forms the initial NO_*x*_ pool in the atmosphere (i-NO_*x*_) (Supplementary Fig. 1). In reality, it is extremely difficult if not impossible to directly measure the i-NO_*x*_ pool due to instantaneous emissions and oxidations. However, the δ^15^N of the i-NO_*x*_ (i.e., δ^15^N_i-NO*x*_) is a straightforward parameter to integrate initial NO_*x*_ emissions and thus to differentiate relative contributions between fossil and non-fossil fuel NO_*x*_ emissions^26,27^. In the atmosphere, the i-NO_*x*_ is partially oxidized to HNO_3_ and particulate NO_3_^−^ (p-NO_3_^−^) (Supplementary Fig. 1), during which N isotopic fractionations^29,30^ lead to substantial δ^15^N differences between ambient NO_*x*_, HNO_3_, and p-NO_3_^−^. Because of the difficulty in constraining δ^15^N differences among these species, it remains a big challenge to evaluate i-NO_*x*_ sources based on δ^15^N signatures of ambient NO_*x*_, HNO_3_, and p-NO_3_^−^. However, precipitation can scavenge both the ambient NO_2_ and the oxidized NO_2_ (i.e., HNO_3_ and p-NO_3_^−^) (Supplementary Fig. 1)^31^. Therefore, we can reconstruct the corresponding δ^15^N_i-NO*x*_ values of the observed δ^15^N_w-NO3−_ values (Supplementary Figs. 1, 2). Assuming that the estimated δ^15^N_i-NO*x*_ value represents and integrates the emission δ^15^N_i-NO*x*_ value, we can differentiate relative contributions between fossil fuel and non-fossil fuel NO_*x*_ emissions^7,24,32^.

Based on the above isotope theory, the δ^15^N_i-NO*x*_ value can be estimated by the following equation (Eq. (1)):

1 $$	\delta ^{15}{\mathrm{N}}_{{\mathrm{i}} - {\mathrm{NOx}}}\\ 	 = (\delta ^{15}{\mathrm{N}}_{{\mathrm{NOx}}} \times {\mathrm{C}}_{{\mathrm{NO2}}}/f_{{\mathrm{NO2}}} + \delta ^{15}{\mathrm{N}}_{{\mathrm{HNO3}}} \times {\mathrm{C}}_{{\mathrm{HNO3}}} + \delta ^{15}N_{{\mathrm{p}} - {\mathrm{NO3}} - } \times {\mathrm{C}}_{{\mathrm{p}} - {\mathrm{NO3}} - }) /\\ 	\hskip 12pt({\mathrm{C}}_{{\mathrm{NO2}}}/f_{{\mathrm{NO2}}} + {\mathrm{C}}_{{\mathrm{HNO3}}} + {\mathrm{C}}_{{\mathrm{p}} - {\mathrm{NO3}} - }),$$

where C_NO2_, C_HNO3_, and C_p-NO3−_ are concentrations of ambient NO_2_, HNO_3_, and p-NO_3_^−^ in the atmosphere, respectively. *f*_NO2_ is the fraction of NO_2_ in NO_*x*_. δ^15^N_NO*x*_, δ^15^N_HNO3_, and δ^15^N_p-NO3−_ are δ^15^N values of NO_*x*_, HNO_3_, and p-NO_3_^−^ in the atmosphere, respectively. The values used for C_NO2_, C_HNO3_, C_p-NO3−_, *f*_NO2_, δ^15^N_NO*x*_, δ^15^N_HNO3_, and δ^15^N_p-NO3−_ are listed in Supplementary Table 1. Due to the limited availability of *f*_NO2_ and δ^15^N_NO*x*_ values, global mean values were used in our calculations (*f*_NO2_ = 64 ± 10%, and δ^15^N_NO*x*_ = −7.7 ± 2.9‰) (Supplementary Table 1).

**Fig. 1:**
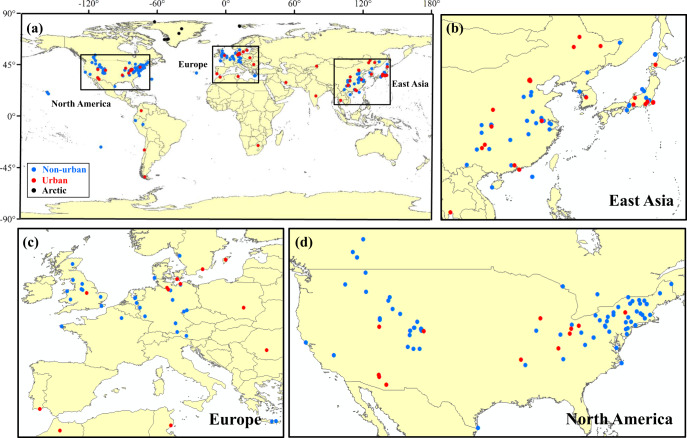
Study sites with δ^15^N_w-NO3−_ observations. Red, blue, and black dots represent urban sites (*n* = 56), non-urban sites (*n* = 158), and Arctic sites (*n* = 8), respectively.

To investigate the importance of non-fossil fuel NO_*x*_ emissions to total NO_*x*_ emissions, we compiled available δ^15^ N values of NO_3_^−^ in precipitation (denoted as δ^15^N_w-NO3−_ hereafter) at urban and non-urban sites of East Asia, Europe, and North America (detailed in “Methods”) (Fig. 1). Both the concentrations and δ^15^N values of ambient NO_*x*_, HNO_3_, and p-NO_3_^−^ were used to constrain the δ^15^N values of the initial mixture of fossil fuel and non-fossil fuel NO_*x*_ in the atmosphere (denoted as δ^15^N_i-NO*x*_, detailed in “Methods”) (Supplementary Fig. 1). Then we evaluated the differences between δ^15^N_w-NO3−_ and δ^15^N_i-NO*x*_ values (denoted as ^15^∆_i-NO*x*→w-NO3−_, ^15^∆_i-NO*x*→w-NO3−_ = δ^15^N_w-NO3−_ - δ^15^N_i-NO*x*_, detailed in “Methods”). By combining the ^15^∆_i-NOx→w-NO3−_ values (Supplementary Fig. 2), the observed δ^15^N_w-NO3−_ values, and δ^15^N values of dominant fossil fuel and non-fossil fuel NO_*x*_ sources (Supplementary Figs. 3, 13), we calculated relative contributions of dominant fossil fuel and non-fossil fuel NO_*x*_ by using a statistical isotope mass-balance model.

## Results and discussion

### Spatial and temporal variations of δ^15^N_w-NO3−_ values

**Fig. 2:**
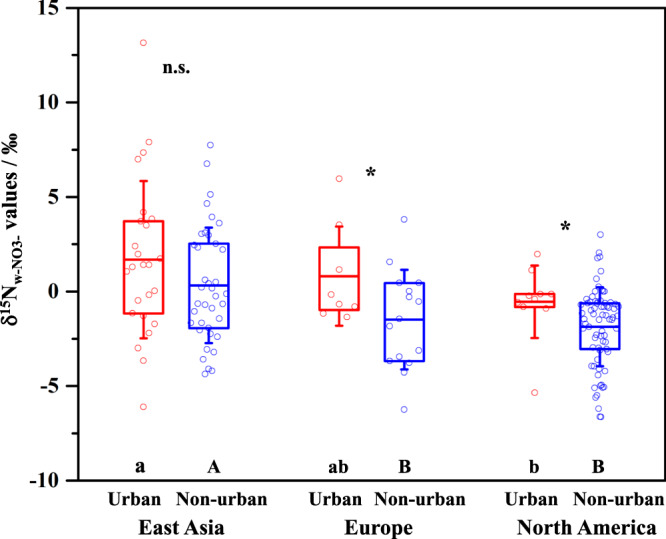
δ^15^N_w-NO3−_ values at urban and non-urban sites of East Asia (*n* = 25 and *n* = 38, respectively), Europe (*n* = 8 and *n* = 15, respectively), and North America (*n* = 10 and *n* = 73, respectively). Dots show mean values of replicate measurements at each site. The box encompasses the 25th−75th percentiles, whiskers, and line in each box are the SD and mean values, respectively. The symbol of * indicates differences between urban and non-urban sites. n.s.: not significant. Different letters indicate differences among East Asia, Europe, and North America. The significance level was set at *P* < 0.1.

In general, East Asia has significantly higher δ^15^N_w-NO3−_ values (1.7 ± 5.4‰ at urban sites and 0.3 ± 3.1‰ at non-urban sites) than Europe (0.8 ± 2.6‰ and −1.5 ± 2.6‰, respectively) and North America (−0.5 ± 1.9‰ and −1.9 ± 2.1‰, respectively) (Fig. 2). This result reflects more influences of the ^15^N-enriched NO_*x*_ from coal combustion (δ^15^N = 13.7 ± 3.9‰; Supplementary Fig. 3) in East Asia than in the other two study regions. Supportively, the amount of coal consumption in East Asia accounted for about 55% of the world’s total amount during 1965–2015, even up to about 64% during 1990–2015 (Supplementary Fig. 4a). Moreover, the NO_*x*_ from coal combustion has influenced δ^15^N_w-NO3−_ signatures of both urban and non-urban areas in East Asia, so that δ^15^N_w-NO3−_ values did not differ between urban and non-urban sites (Fig. 2). The δ^15^N_w-NO3−_ values are lower at non-urban sites than at urban sites in Europe and North America (Fig. 2), reflecting more influences of the ^15^N-depleted NO_*x*_ from microbial N cycle (δ^15^N = −30.2 ± 6.7‰; Supplementary Fig. 3) at non-urban sites of these two regions than that of East Asia.

**Fig. 3:**
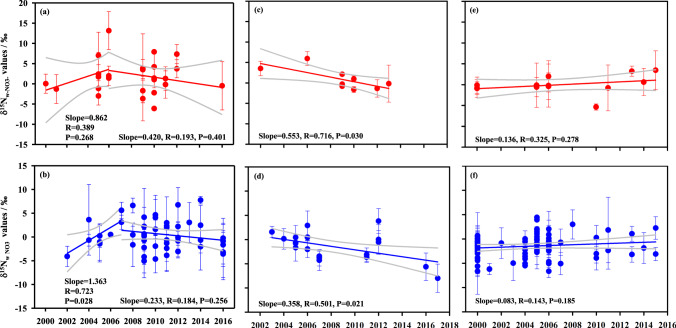
Variations of δ^15^N_w-NO3−_ values during 2000–2017. **a**, **b** Urban and non-urban sites of East Asia. **c**, **d** Urban and non-urban sites of Europe. **e**, **f** Urban and non-urban sites of North America. Mean values of replicate measurements at each site in each year are shown. The gray lines are the 95% confidence intervals.

The three study regions exhibit different temporal variations in δ^15^N_w-NO3−_ values (Fig. 3). In East Asia, δ^15^N_w-NO3−_ values increased at both urban and non-urban sites from 2000 to 2007 and then decreased very slowly (Fig. 3). This trend reflects the controlling strategies of NO_*x*_ emissions from coal combustion in East Asia, particularly in China. During 2000–2007, the amount of coal consumption in China accounts for 89 ± 2% of the total amount in East Asia (Supplementary Fig. 4b). As a turning point, China started to implement mitigation measures for NO_*x*_ from coal combustion in 2007, i.e., the policy of “replacing small generation units with large ones” for coal power plants^33,34^. Since 2008, a large-scale flue gas denitrification technology has been widely utilized in coal-fired power plants of China to reduce the NO_*x*_ emission from industrial coal combustion^33,35^. Differently, δ^15^N_w-NO3−_ values in Europe decreased from 2002 to 2017 (Fig. 3) in response to a decrease in NO_*x*_ emissions from the coal combustion because the coal consumption in Europe has reduced by 20% from 2002 to 2017 (Supplementary Fig. 4a). Although there was a significant decrease in the amount of coal combustion (by 34%) in North America during 2000–2017 (Supplementary Fig. 4a), corresponding δ^15^N_w-NO3−_ values were relatively consistent (Fig. 3). This pattern reflects the NO_*x*_ emission reduction technology used in power plants because the technology can raise δ^15^N values of NO_*x*_ emitted^36^.

### Importance of non-fossil fuel NO_*x*_ emissions

**Fig. 4:**
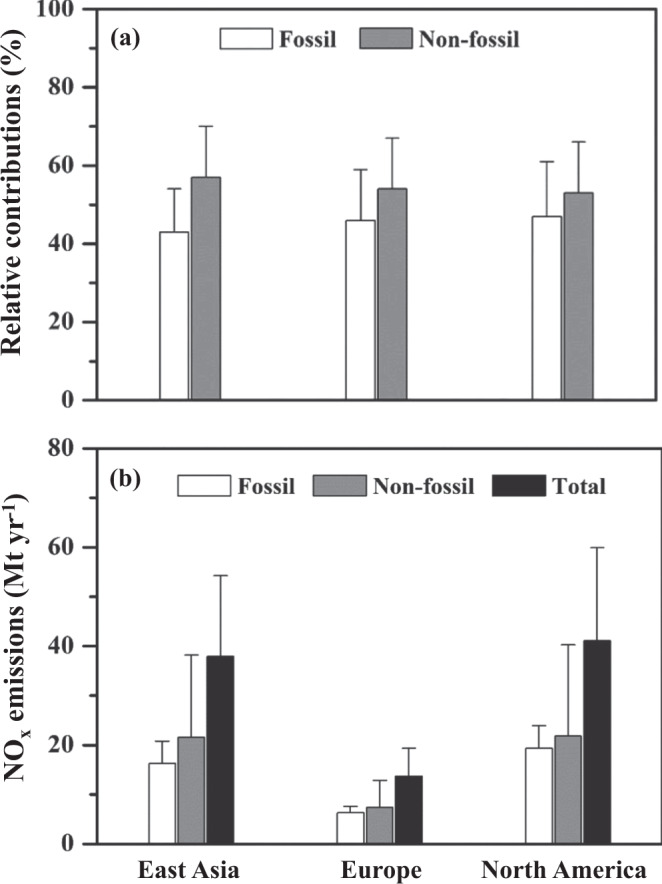
Fossil and non-fossil fuel NO_x_ emissions in East Asia, Europe, and North America. **a** Relative contributions. **b** Emission amounts. Mean ± SD values are shown.

Results from the Stable Isotope Analysis in R (the SIAR model; detailed in “Methods”) showed that variations in relative contributions of NO_*x*_ from coal combustion are the main cause of different temporal patterns of regional δ^15^N_w-NO3−_ variations. (Supplementary Figs. 5–8). However, relative contributions of non-fossil fuel NO_*x*_ emissions average 49 ± 11% at urban sites and 69 ± 13% at non-urban sites for all three study regions (Supplementary Fig. 9). By integrating urban and non-urban sites in each region, we found that relative contributions of non-fossil fuel NO_*x*_ average 57 ± 13% in East Asia, 54 ± 13% in Europe, and 53 ± 13% in North America (Fig. 4a, Supplementary Fig. 9). Based on mean annual emission amounts of NO_*x*_ from coal combustion and vehicle exhausts (Fig. 4b, Supplementary Fig. 10) and their annual mean relative contributions to total NO_*x*_ emissions (Fig. 4a), the mean annual NO_*x*_ emissions are estimated (detailed in “Methods”) at 37.9 ± 16.4Mt yr^−1^ in East Asia during 2000–2016, 13.7 ± 5.6Mt yr^−1^ in Europe during 2000–2017, and 41.1 ± 18.8Mt yr^−1^ in North America during 2000–2015, respectively (Fig. 4b). Then, non-fossil fuel NO_*x*_ emission has been determined at 21.6 ± 16.6Mt yr^−1^ in East Asia, 7.4 ± 5.5Mt yr^−1^ in Europe, and 21.8 ± 18.5Mt yr^−1^ in North America, respectively (Fig. 4b). These values for regional NO_*x*_ emissions are valuable because they have long been missing in budgeting NO_*x*_ deposition and modeling effects of atmospheric NO_*x*_ loading.

Although we have considered uncertainties, there are still a few factors that remain difficult to quantify in the current stage. First, not all NO_*x*_ emission sources have been considered in δ^15^N observations, and other sources such as natural gases and oil fuel combustion might be important in a few sites. Second, data heterogeneities in time and space are also a source of uncertainty, as it is almost impossible to measure the parameters used in our calculations simultaneously. Furthermore, the SIAR model only provides possible distributions but not definitive solutions of relative contributions of multiple sources. Therefore, future efforts on constraining these uncertainties will improve natural isotope evidence on global NO_*x*_ emissions.

### Remarks

Our study provides direct isotope evidence on that the changes in regional human activities have distinct influences on δ^15^N signatures of deposited NO_x_ to terrestrial environments. The δ^15^N_w-NO3−_ values exhibit significant spatiotemporal changes, which can be used to trace anthropogenic N inputs and help us understand decadal δ^15^N variations in materials of surface–earth systems, such as tree rings, sediments, and oceanic biota. Currently, environmental policies in many countries of the study regions mostly aim to mitigate more fossil fuel NO_*x*_ emissions via technology promotion and energy structure adjustment. However, our study shows that non-fossil fuel NO_*x*_ emission is equally as important as fossil fuel NO_x_ emission, and it has long been underestimated. Accordingly, the control of non-fossil fuel NO_*x*_ emissions should be equally considered in the mitigation of NO_*x*_ pollution. Moreover, regional NO_*x*_ emissions newly constrained in this study are useful for budgeting NO_3_^−^ deposition fluxes and modeling ecological and climatic effects of atmospheric NO_3_^−^ loading.

## Methods

### Global δ^15^N_w-NO3−_ observations

Publications of δ^15^N_w-NO3−_ studies were obtained through the databases of the Web of Science (http://isiknowledge.com), Google Scholar (http://scholar.google.com.hk), and Baidu Scholar (http://xueshu.baidu.com) by searching keywords of “nitrogen isotope”, “nitrate”, “rainfall”, and “precipitation”. By the end of December 2018, a total of 128 publications were available (Supplementary Text 1), spanning the sampling time of 1956–2017 (Supplementary Fig. 11). We extracted δ^15^N_w-NO3−_ values of individual precipitation samples by using the software of Web Plot Digitizer^37^.

There are totally 3483 individual δ^15^N_w-NO3−_ data and 222 sampling sites when multiple observations in different sampling years at the same site were counted once only (Fig. 1). There are 56 urban sites, 158 non-urban sites, and eight arctic sites (Fig. 1), in which non-urban sites are mainly situated in rural, mountain, forest, and lake areas. Due to the sparsity of available data before 2000 (Supplementary Fig. 11), we analyzed δ^15^N_w-NO3−_ data at major urban and non-urban sites in East Asia, Europe, and North America during 2000–2017 to ensure a better site representation and to reduce the uncertainty caused by inconsistency in sampling time (Fig. 1). To describe spatial differences in δ^15^N_w-NO3−_ values between urban and non-urban sites among three regions (totally 214 sites), only site-based mean values during the period of 2000–2017 (totally 169 sites) were used (detailed in Fig. 2). To describe temporal variations of δ^15^N_w-NO3−_ values in urban and non-urban areas of each region, respectively (Fig. 3), we counted observation sites by different sampling years, given that δ^15^N_w-NO3−_ observations at few sites have been conducted in different sampling years. In this way, there were a total of 206 sites during 2000–2017 (detailed in Fig. 3). In addition, 35%, 29%, and 36% of the δ^15^N_w-NO3−_ observations were conducted in warmer, cooler, and the whole year, respectively. The seasonal effects of NO_*x*_ emissions may not substantially influence the patterns of regional δ^15^N_w-NO3−_ variations.

### Differences between δ^15^N_w-NO3−_ and δ^15^N_i-NO*x*_ values

NO is normally insoluble in water, and w-NO_3_^−^ is scavenged only from the ambient NO_2_ and the oxidized NO_*x*_ (i.e., HNO_3_ and p-NO_3_^−^) (Supplementary Fig. 1)^32,38,39^. Moreover, isotopic effects during the NO_*x*_ cycles lead to differences between δ^15^N_NO*x*_ and δ^15^N_NO2_. Therefore, substantial differences exist between the δ^15^N_w-NO3−_ and δ^15^N_i-NO*x*_ values in the atmosphere (hereafter denoted as ^15^∆_i-NO*x*→w-NO3−_). In this study, we calculated ^15^∆_i-NO*x*→w-NO3−_ values by using the following equation (Eq. (2)):

2 $${\,}^{15}{\Delta}_{{\mathrm{i}} - {\mathrm{NO}x} \to {\mathrm{w}} - {\mathrm{NO3}} - } = \delta ^{15}{\mathrm{N}}_{{\mathrm{w}} - {\mathrm{NO3}} - } - \delta ^{15}{\mathrm{N}}_{{\mathrm{i}} - {\mathrm{NO}x}}.$$

Combined Eq. (1) with Eq. (2), we get Eq. (3) to calculate the ^15^∆_i-NO*x*→w-NO3−_ values.

3 $$	{\,}^{15}{\Delta}_{{\mathrm{i}} - {\mathrm{NO}x} \to {\mathrm{w}} - {\mathrm{NO3}}} = \delta ^{15}{\mathrm{N}}_{{\mathrm{w}} - {\mathrm{NO3}} - }\\ 	 \quad- \left({\delta}^{15}{\mathrm{N}}_{{\mathrm{NO}x}} \times {\mathrm{C}}_{{\mathrm{NO2}}}/f_{{\mathrm{NO2}}} + \delta ^{15}{\mathrm{N}}_{{\mathrm{HNO3}}} \times {\mathrm{C}}_{{\mathrm{HNO3}}} + \delta ^{15}{\mathrm{N}}_{{\mathrm{p}} - {\mathrm{NO3}} - } \times {\mathrm{C}}_{{\mathrm{p}} - {\mathrm{NO3}}}\right)/\\ 	 \quad \left({\mathrm{C}}_{{\mathrm{NO2}}}/f_{{\mathrm{NO2}}} + {\mathrm{C}}_{{\mathrm{HNO3}}} + {\mathrm{C}}_{{\mathrm{p}} - {\mathrm{NO3}} - }\right).$$

To obtain more accurate ^15^∆_i-NO*x*→w-NO3−_ values, we estimated the ^15^∆_i-NO*x*→w-NO3_− values in two independent scenarios. In Scenario 1, mean values of global δ^15^N_NO*x*_ and *f*_NO2_ values, simultaneously observed values of ambient C_NO2_, C_HNO3_, C_p-NO3−_, δ^15^N_HNO3_, δ^15^N_p-NO3−_, and δ^15^N_w-NO3−_ were used for the calculation in Eq. (3). In Scenario 2, non-synchronously observed values of ambient *f*_NO2_, C_NO2_, C_HNO3_, C_p-NO3−_, δ^15^N_NO*x*_, δ^15^N_HNO3_, δ^15^N_p-NO3−_, and δ^15^N_w-NO3−_ were used for the calculation in Eq. (3). The values and data sources of parameters used for estimating ambient ^15^∆_i-NO*x*→w-NO3−_ values are included in Supplementary Table 1. Because data of *f*_NO2_ and δ^15^N_NO*x*_ are very sparse globally, we used global mean values and considered their SD values into the uncertainty analysis by the Monte Carlo method. Furthermore, because of no significant difference between ^15^∆_i-NO*x*→w-NO3−_ values obtained in Scenario 1 (2.1 ± 1.7‰) and Scenario 2 (5.7 ± 3.2‰) (Supplementary Fig. 2), we used a mean value of them (3.9 ± 1.8‰; Supplementary Fig. 2) in the calculations of source contributions (Eqs. (4) and (5)).

### Contributions of dominant fossil fuel and non-fossil fuel NO_*x*_ sources

Based on δ^15^N_w-NO3−_, ^15^∆_i-NO*x*→w-NO3−_, and δ^15^N values of NO_*x*_ sources, we estimated relative contributions of dominant fossil fuel and non-fossil fuel NO_*x*_ sources to total NO_*x*_ emissions by using the isotope mass-balance method. We considered coal combustion (denoted as S1) and vehicle exhausts (S2) as dominant fossil fuel NO_x_ sources, and biomass burning (S3), and microbial N cycles (S4) as dominant non-fossil fuel NO_*x*_ sources. The major reasons include: (1) these four sources have been considered as dominant sources of total NO_*x*_ emissions in studies of both emission inventory and deposition modeling^2,9,11,13–15,19–21^; (2) they are also the dominant sources influencing δ^15^N variations of NO_*x*_ and NO_3_^−^ in the atmosphere;^26,27^ (3) their mean δ^15^N values of NO_*x*_ emission sources differ significantly (*P* < 0.05, Supplementary Fig. 3) and therefore can be used to differentiate their relative contributions.

The S1–S4 are considered as dominant NO_*x*_ sources at urban sites but S2 cannot be considered as a dominant NO_*x*_ source at non-urban sites. First of all, studies of roadside NO_*x*_ emissions have evidenced that vehicle exhausts contribute little to atmospheric NO_*x*_ at non-urban sites due to limited amounts of long-range transport^40–42^. Statistical data also show 76%, 82%, and 78% of vehicles distributed in urban areas of East Asia, North America, and Europe, respectively while their urban areas account for only 1.7%, 1.4%, and 16.6% of total land area, respectively (Supplementary Tables 2, 3, Supplementary Fig. 12). Secondly, 76% and 91% of δ^15^N_w-NO3−_ values at urban and non-urban sites fall in the δ^15^N range of NO_*x*_ from vehicle exhausts (Supplementary Figs. 3, 13). Consequently, when the NO_*x*_ from vehicle exhausts is considered into the calculations of relative contributions of different NO_*x*_ sources at non-urban sites, its contributions at non-urban sites (25 ± 12%) are similar to urban sites (28 ± 8%), which is unlikely. Besides, because mutual NO_x_ transportations always occur between urban and non-urban areas, δ^15^N values of NO_3_^−^ in precipitation at a given urban or non-urban site integrate δ^15^N values of NO_*x*_ from both local emissions and regional transportations. However, physical NO_*x*_ transportation might have no substantial isotope effects, and thus likely will not influence the site-specific evaluations of fossil and non-fossil fuel NO_*x*_ contributions.

According to isotope mass-balance theory, we calculated relative contributions of S1–S4 (*f*_S1_, *f*_S2_, *f*_S3_, and *f*_S4_, respectively) at urban sites by using Eq. (4):

4 $$\delta ^{15}{\mathrm{N}}_{{\mathrm{w}} - {\mathrm{NO3}} - } =	 (f_{{\mathrm{S1}}} \times \delta ^{15}{\mathrm{N}}_{{\mathrm{S1}}} + f_{{\mathrm{S2}}} \times \delta ^{15}{\mathrm{N}}_{{\mathrm{S2}}} + f_{{\mathrm{S3}}} \times \delta ^{15}{\mathrm{N}}_{{\mathrm{S3}}} + f_{{\mathrm{S4}}} \times \delta ^{15}{\mathrm{N}}_{{\mathrm{S4}}})\\ 	+ {\,}^{15}{\Delta}_{{\mathrm{i}} - {\mathrm{NOX}} \to {\mathrm{w}} - {\mathrm{NO3}} - },$$

where we assumed that *f*_S1_ + *f*_S2_ + *f*_S3_ + *f*_S4_ = 1.

Then, we calculated their relative contributions at non-urban sites by Eq. (5):

5 $$\delta ^{15}{\mathrm{N}}_{{\mathrm{w}} - {\mathrm{NO3}} - } =	 {\,\,} (f_{{\mathrm{S1}}} \times \delta ^{15}{\mathrm{N}}_{{\mathrm{S1}}} + f_{{\mathrm{S3}}} \times \delta ^{15}{\mathrm{N}}_{{\mathrm{S3}}} + f_{{\mathrm{S4}}} \times \delta ^{15}{\mathrm{N}}_{{\mathrm{S4}}})\\ 	+ {\,}^{15}{\Delta}_{{\mathrm{i}} - {\mathrm{NO}X} \to {\mathrm{w}} - {\mathrm{NO3}} - },$$

where we assumed that *f*_S1_ + *f*_S3_ + *f*_S4_ = 1. δ^15^N_S1_, δ^15^N_S2_, δ^15^N_S3_, and δ^15^N_S4_ represent δ^15^N values of NO_*x*_ from coal combustion (S1), vehicle exhausts (S2), biomass burning (S3), and microbial N cycles (S4), respectively (Supplementary Fig. 3).

The *f*_S1_, *f*_S2_, *f*_S3_, and *f*_S4_ values were calculated by using a Bayesian isotope-mixing model (named Stable Isotope Analysis in R, SIAR). The SIAR model^43^ uses a Bayesian framework to establish a logical prior distribution based on Dirichlet distribution^44^ for estimating source contributions (*f*_S1_–*f*_S4_). It has the potential to provide reliable estimations of source contributions because the isotope effect (i.e., ^15^∆_i-NO*x*→w-NO3−_ values in this study), the variability in δ^15^N values of both sources (i.e., δ^15^N values of NO_*x*_ from S1–S4 in this study), and the mixture (i.e., δ^15^N_w-NO3−_ values in this study)^45,46^ are considered. The SIAR model has been widely used to quantify the relative contributions of multiple NO_*x*_ emission sources to p-NO_3_^−^ and w-NO_3_^−^^26,27,31,47^. In each run of the SIAR model, the mean ± SD of δ^15^N_NO*x*_ values (Supplementary Fig. 3), the mean ± SD of ^15^∆ _w-NO3−→i-NO*x*_ values (Supplementary Fig. 2), and replicate δ^15^N_w-NO3−_ values at each urban or non-urban site in each sampling year (Fig. 3) were input into the model. In addition, the percentage data of each source (*n* = 10,000) output from each run of the SIAR model were used to calculate mean ± SD values of corresponding source contributions (Supplementary Figs. 5–8).

We calculated the total contribution of each NO_*x*_ source in each region (*F*; Eq. (6)) by using its annual mean relative contributions at urban and non-urban sites during 2000–2017 (*n* = 28, 9, 13 for urban sites and *n* = 47, 21, 88 for non-urban sites in East Asia, Europe, and North America, respectively) (*f*_urban_ and *f*_non-urban_, respectively; Supplementary Fig. 9) and annual mean proportions of urban and non-urban populations in the total population of each region during 2000–2017 (*P*_urban_ and *P*_non-urban_, respectively; Supplementary Fig. 14).

6 $${{F}} = f_{{\mathrm{urban}}} \times P_{{\mathrm{urban}}} \times f_{{\mathrm{non - urban}}} \times P_{{\mathrm{non - urban}}}.$$

Then, we calculated annual mean relative contributions of dominant fossil fuel and non-fossil fuel NO_*x*_ sources in each region (*F*_fossil_ and *F*_non-fossil_, respectively) by using Eq. (7) and Eq. (8), respectively.

7 $$F_{{\mathrm{fossil}}} = F_{{\mathrm{S1}}} + F_{{\mathrm{S2}}},$$

8 $$F_{{\mathrm{non - fossil}}} = F_{{\mathrm{S3}}} + F_{{\mathrm{S4}}}.$$

Finally, based on the annual mean amounts of fossil fuel NO_*x*_ emissions (*A*_fossil_) in East Asia during 2000–2010, in Europe during 2000–2015, and in North America during 2000–2016, respectively (Fig. 4b, Supplementary Fig. 10), the annual mean amounts of total NO_*x*_ emissions (*A*_total_) and non-fossil fuel NO_*x*_ emissions (*A*_non-fossil_) in each region during 2000–2017 were calculated by using Eq. (9) and Eq. (10), respectively:

9 $${{A}}_{{\mathrm{total}}} = {{A}}_{{\mathrm{fossil}}}/F_{{\mathrm{fossil}}},$$

10 $${{A}}_{{\mathrm{non - fossil}}} = {{A}}_{{\mathrm{total}}} - {{A}}_{{\mathrm{fossil}}}.$$

We estimated the SD values of calculated values in Eqs. (6)–(10) and finally propagated into the uncertainties of the A_non-fossil_ values by using the Monte Carlo method.

### Statistical analyses

The one-way analyses of variance (Fig. 2) and Pearson correlation analyses (Fig. 3) were performed by using the Origin 2016 statistical package (OriginLab Corporation, USA) and SPSS 16.0 statistical package (SPSS Inc., Chicago, IL). Because of regionally limiting observation sites and inherently high variability of δ^15^N_w-NO3−_, spatial differences are significant only at the level of *P* < 0.1 (Fig. 2). Mean values and standard deviation (SD) were reported.

## Supplementary information

Supplementary Information

## Data Availability

The data underlying the findings of this study are available in this article.  Source data are provided with this paper.

## Source data

Source Data
