# Supplementary material for: Important contributions of non-fossil fuel nitrogen oxides emissions
Source: Nat Commun. 2021 Jan 11;12:243. doi: 10.1038/s41467-020-20356-0 (PMC7801390; doi:10.1038/s41467-020-20356-0)
Supplement: Supplementary file 1 — Supplementary Information [file 41467_2020_20356_MOESM1_ESM.pdf]

Supplementary Information for

Important contributions of non-fossil fuel nitrogen oxides emissions

Wei Song[1], Xue-Yan Liu[1], Chao-Chen Hu[1], Guan-Yi Chen[2], Xue-Jun Liu[3], Wendell W. Walters[4], Greg Michalski[5], Cong-Qiang Liu[1]

1. School of Earth System Science, Tianjin University, Tianjin, 300072, China
2. Georgia Tech Shenzhen Institute, Tianjin University, Shenzhen 518071, China
3. College of Resources and Environmental Sciences, China Agricultural University, Beijing, 100193, China
4. Institute at Brown for Environment and Society, Brown University, 85 Waterman St, Providence, RI, 02912, United States
5. Department of Earth, Atmospheric, and Planetary Sciences, Purdue University, 550 Stadium Mall Drive, West Lafayette, IN 47907, United States

Materials & Correspondence.
Correspondence and requests for materials should be addressed to Xue-Yan Liu (liuxueyan@tju.edu.cn).

This file includes:

1. **Supplementary Figs. 1–14**
2. **Supplementary Tables. 1–3**
3. **Supplementary Note 1:**
    1) Supplementary references 21−148 in Supplementary Text 1 are publications of $\delta^{15}N_{w\text{-}NO3\text{-}}$ observations.
    2) Supplementary references 149–154 in Supplementary Text 2 are publications of simultaneous observations of HNO_3, p-NO_3^-, and NO_2 concentrations in the atmosphere.
    3) Supplementary references 155−157 in Supplementary Text 3 are data sources of observations of HNO_3, p-NO_3^-, and NO_2 concentrations in the ambient atmosphere.
    4) Supplementary references 158−165 in Supplementary Text 4 are publications of observations of ambient $f_{NO2/NOx}$ values.
    5) Supplementary references 166−169 in Supplementary Text 5 are publications of ambient $\delta^{15}N_{NOx}$ observations.
    6) Supplementary references 170−173 in Supplementary Text 6 are publications of simultaneous $\delta^{15}N$ observations of HNO_3, p-NO_3^-, and w-NO_3^- in the atmosphere.
    7) Supplementary references 174−182 in Supplementary Text 7 are publications of ambient $\delta^{15}N_{HNO3}$ observations.
    8) Supplementary references 183−213 in Supplementary Text 8 are publications of $\delta^{15}N_{p\text{-}NO3\text{-}}$ observations.

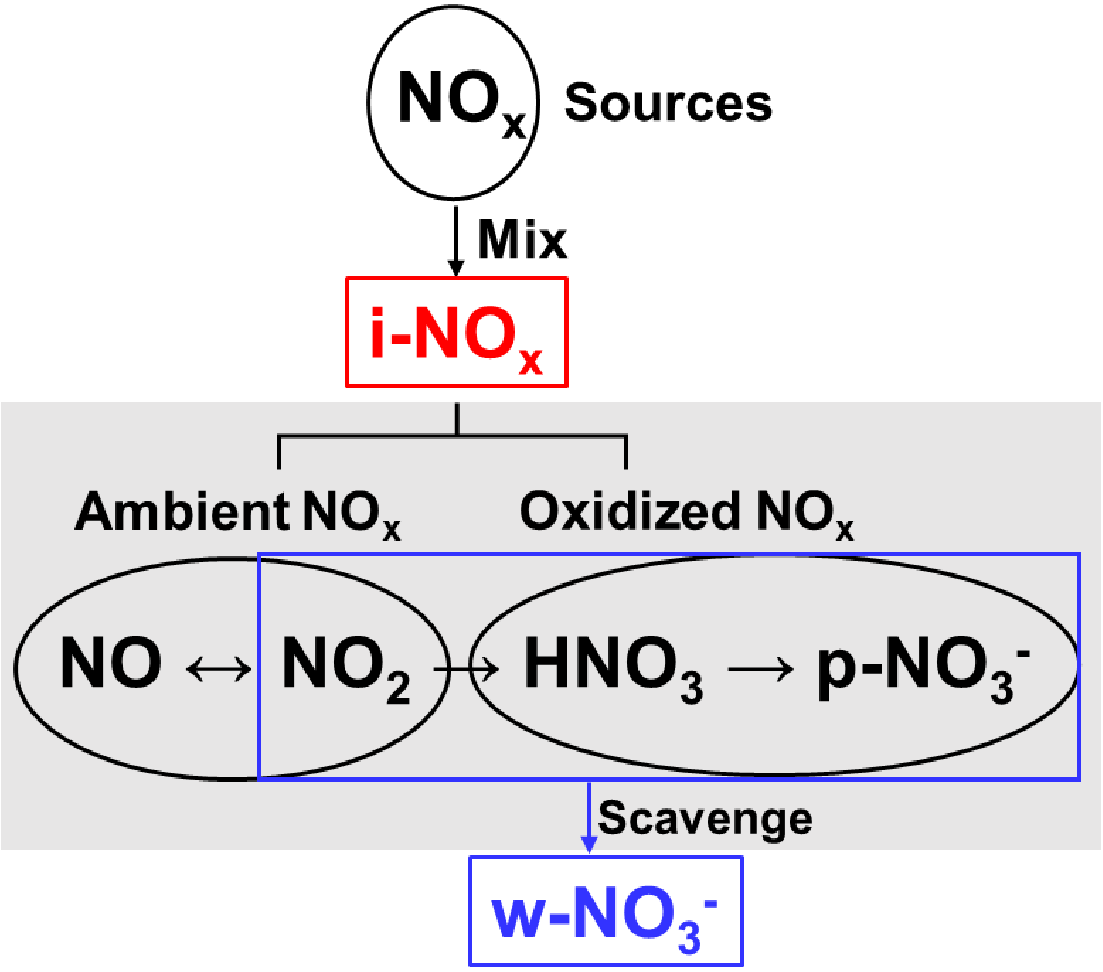

Supplementary Fig. 1 | A schematic map showing relationships between NO_x, HNO_3, p-NO_3^-, and w-NO_3^- in the atmosphere (detailed in Methods)**.**

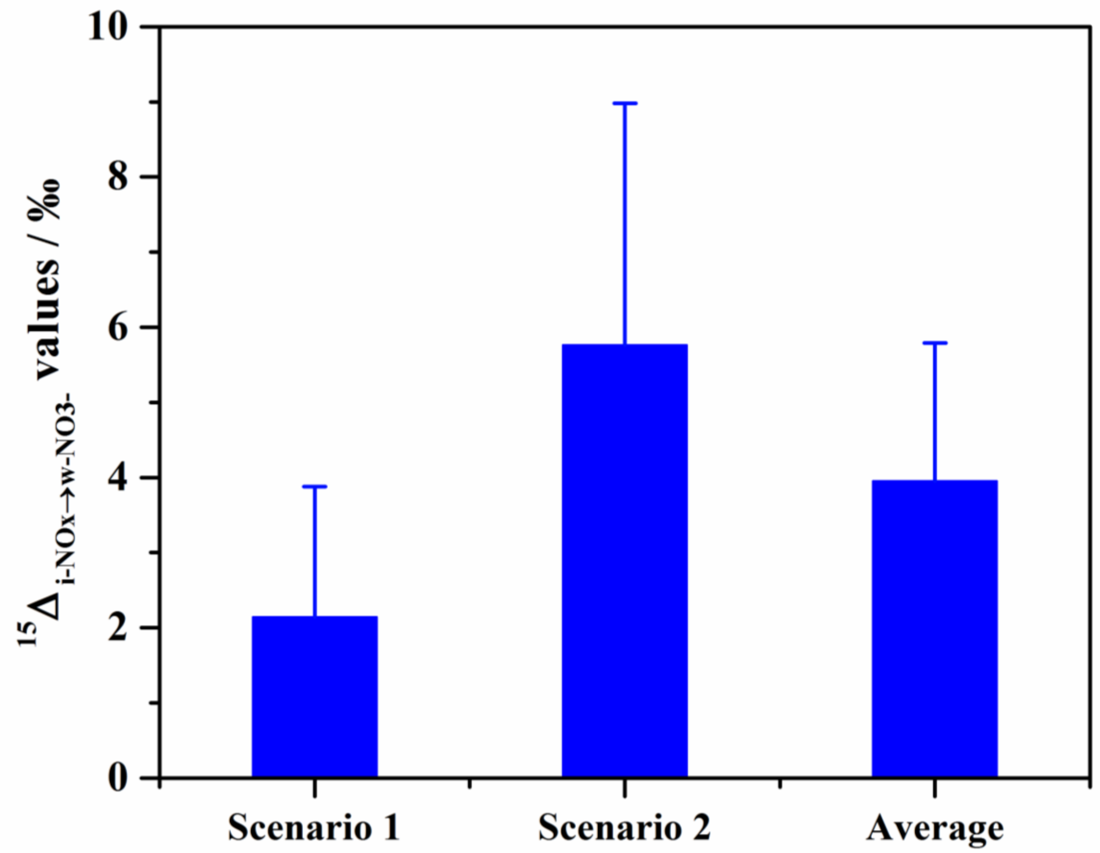

Supplementary Fig. 2 | Differences in δ^{15}N values between w-NO_3^- and i-NO_x in the atmosphere ($^{15}\Delta_{i\text{-}NOx \rightarrow w\text{-}NO3\text{-}}$ values). Mean ± SD values are shown. Scenarios 1 & 2 show estimations based on simultaneous and non-synchronous observations of atmospheric NO_x, HNO_3, p-NO_3^-, and w-NO_3^- (detailed in Methods). Average: the mean value of mean values of Scenarios 1 & 2.

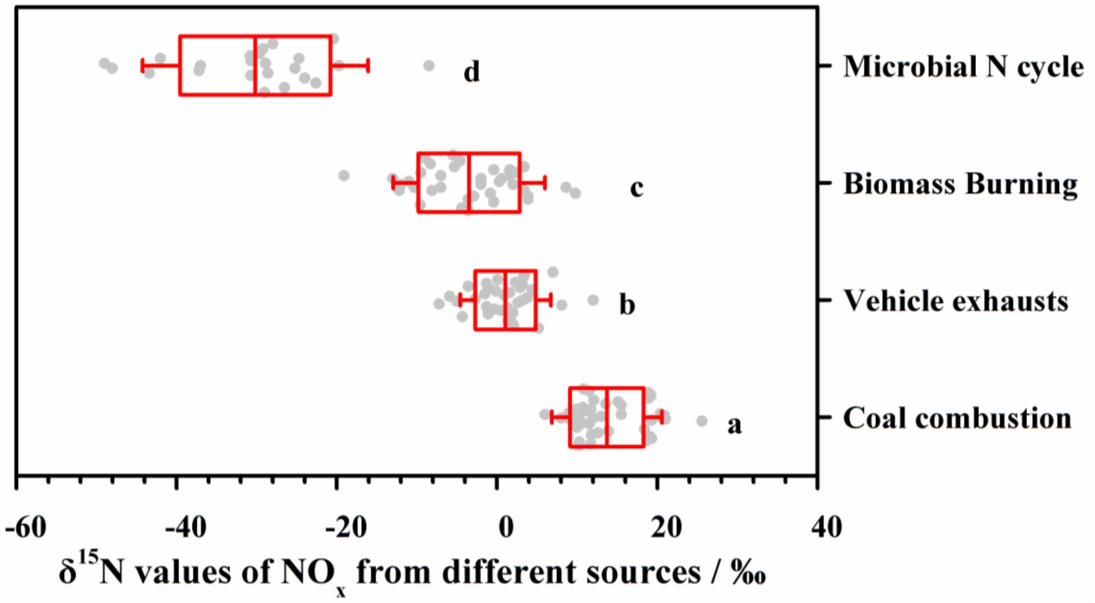

Supplementary Fig. 3 | δ^{15}N values of NO_x from major sources. Dots around each box show scatter values. The box encompasses the 25th–75th percentiles, whiskers and line in each box are the SD and mean values, respectively. Values with different letters are significantly different at $P<0.05$. Data were cited from refs. 1–9.

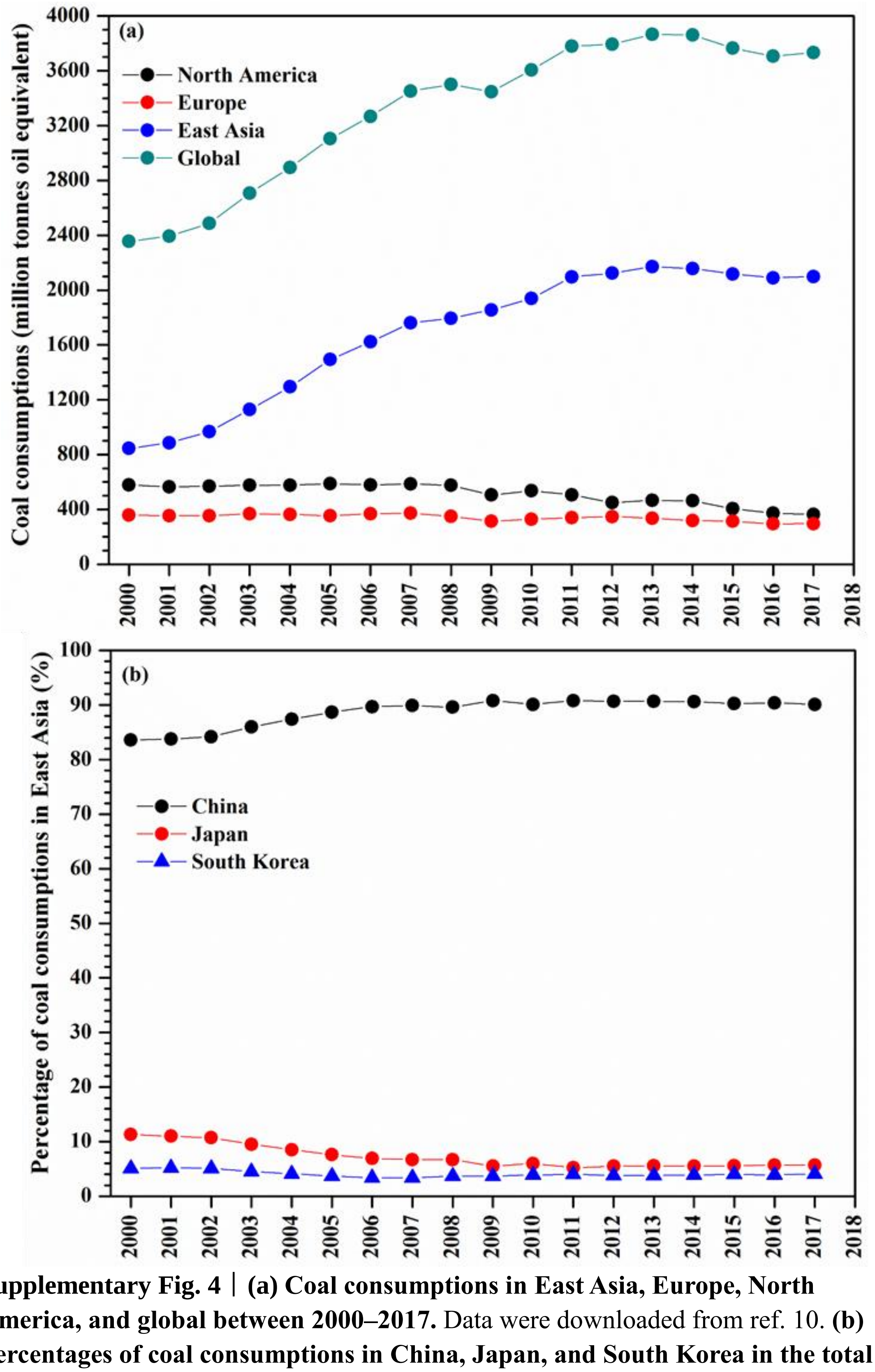

Supplementary Fig. 4 | (a) Coal consumptions in East Asia, Europe, North America, and global between 2000–2017. Data were downloaded from ref. 10. **(b) Percentages of coal consumptions in China, Japan, and South Korea in the total amount of East Asia during 2000–2017.** Data were downloaded from ref. 10.

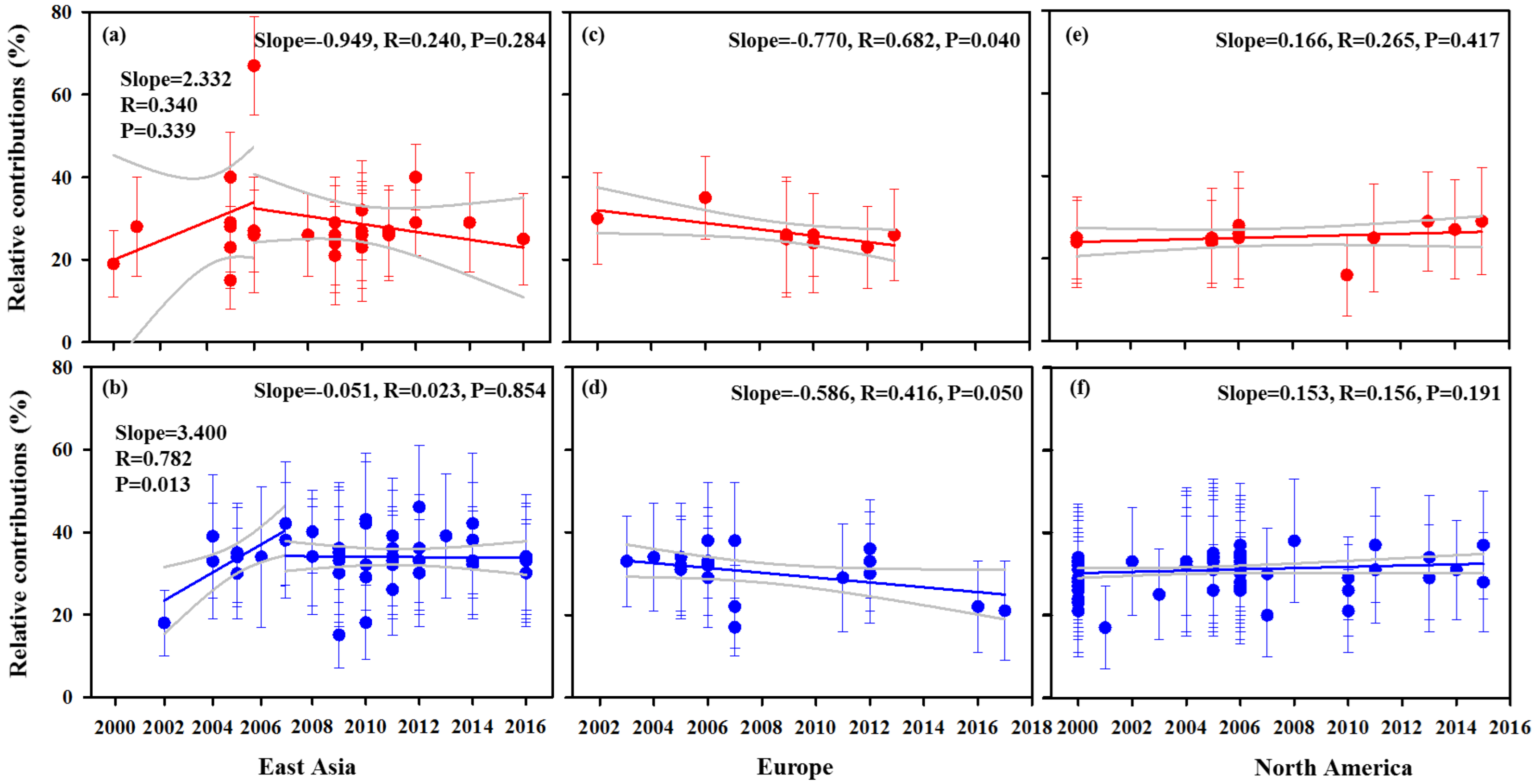

Supplementary Fig. 5 | Variations of relative contributions of NO_x from coal combustion during 2000–2017 at urban (a, c, and e) and non-urban (b, d, and f) sites of East Asia, Europe, and North America, respectively. Mean ± SD values of relative contributions at each site in each year are shown (*n*=28, 9, 13 for panels a, c, e and *n*=47, 21, 88 for panels b, d, f, respectively). Calculations were detailed in Methods. The gray lines were the 95% confidence intervals.

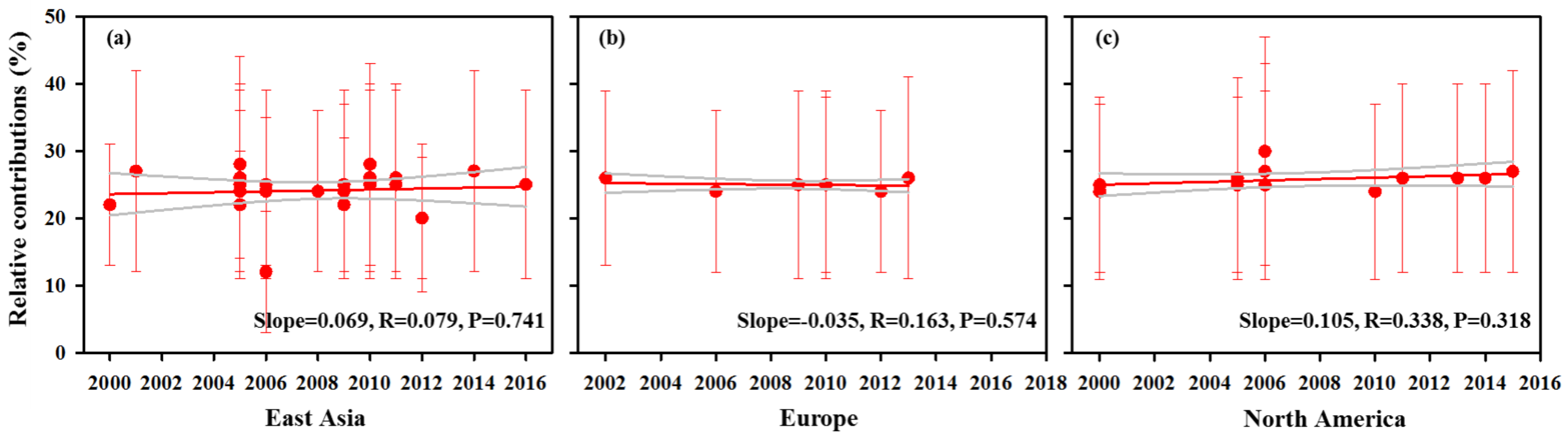

Supplementary Fig. 6 | Variations of relative contributions of NO_x from vehicle exhausts during 2000–2017 at urban (a, b, and c) sites of East Asia, Europe, and North America, respectively. Mean ± SD values of relative contributions at each site in each year are shown (*n*=28, 9, 13 for panels a, b, and c, respectively). Calculations were detailed in Methods. The gray lines were the 95% confidence intervals.

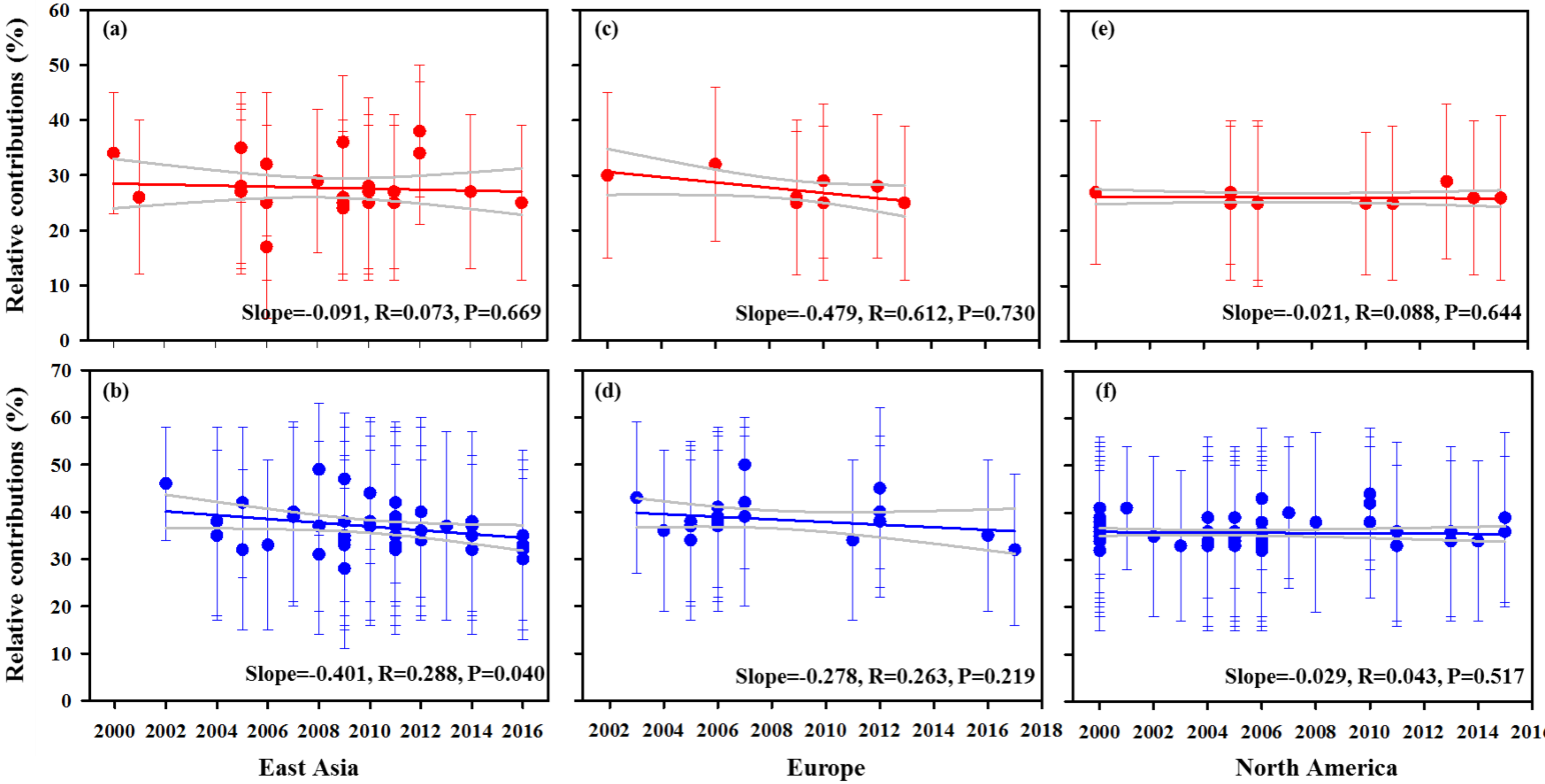

Supplementary Fig. 7 | Variations of relative contributions of NO_x from biomass burning during 2000–2017 at urban (a, c, and e) and non-urban (b, d, and f) sites of East Asia, Europe, and North America, respectively. Mean ± SD values of relative contributions at each site in each year are shown (*n*=28, 9, 13 for panels a, c, e and *n*=47, 21, 88 for panels b, d, f, respectively). Calculations were detailed in Methods. The gray lines were the 95% confidence intervals.

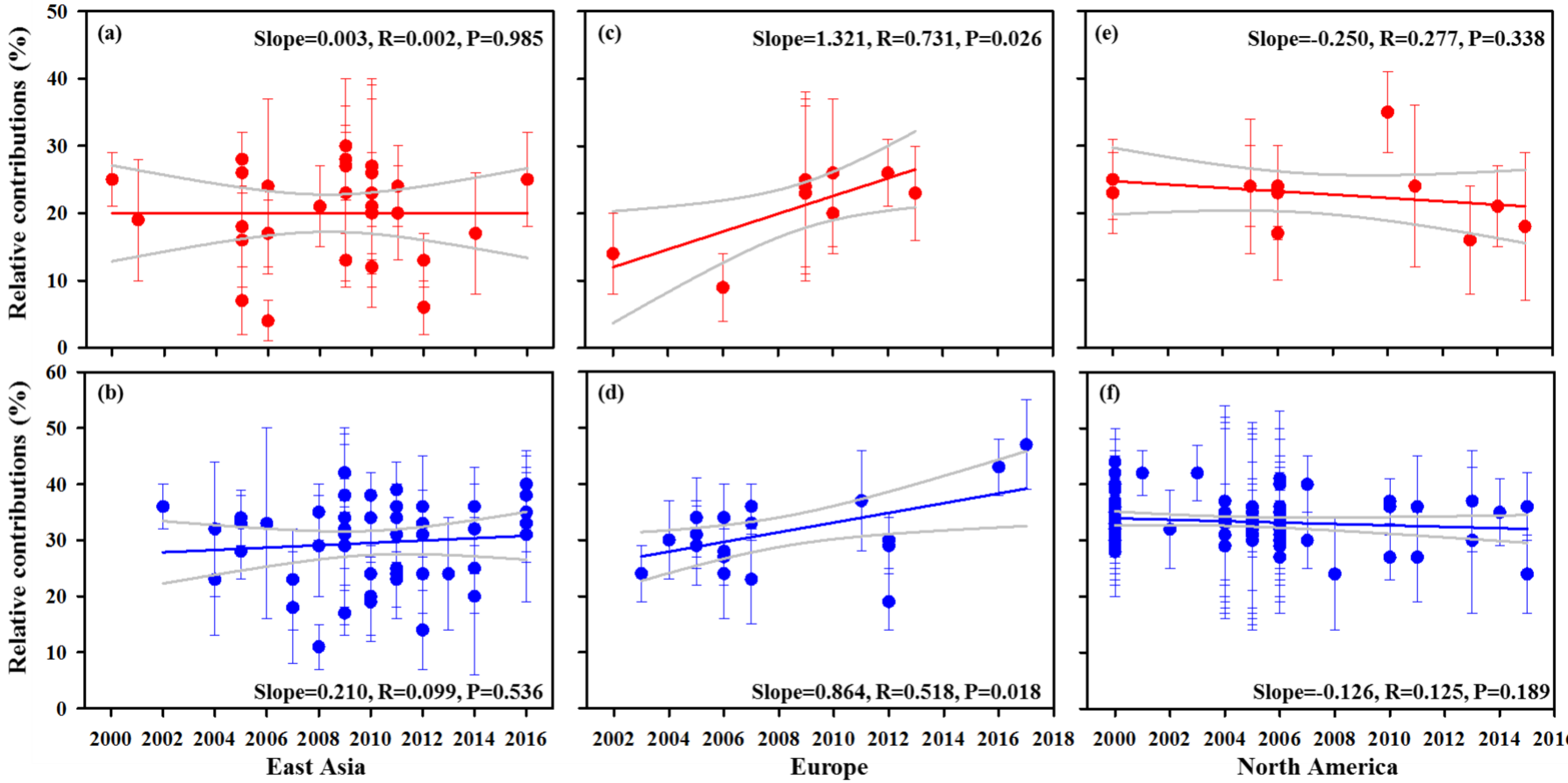

Supplementary Fig. 8 | Variations of relative contributions of NO_x from microbial N cycle during 2000–2017 at urban (a, c, and e) and non-urban (b, d, and f) sites of East Asia, Europe, and North America, respectively. Mean ± SD values of relative contributions at each site in each year are shown (*n*=28, 9, 13 for panels a, c, e and *n*=47, 21, 88 for panels b, d, f, respectively). Calculations were detailed in Methods. The gray lines were the 95% confidence intervals.

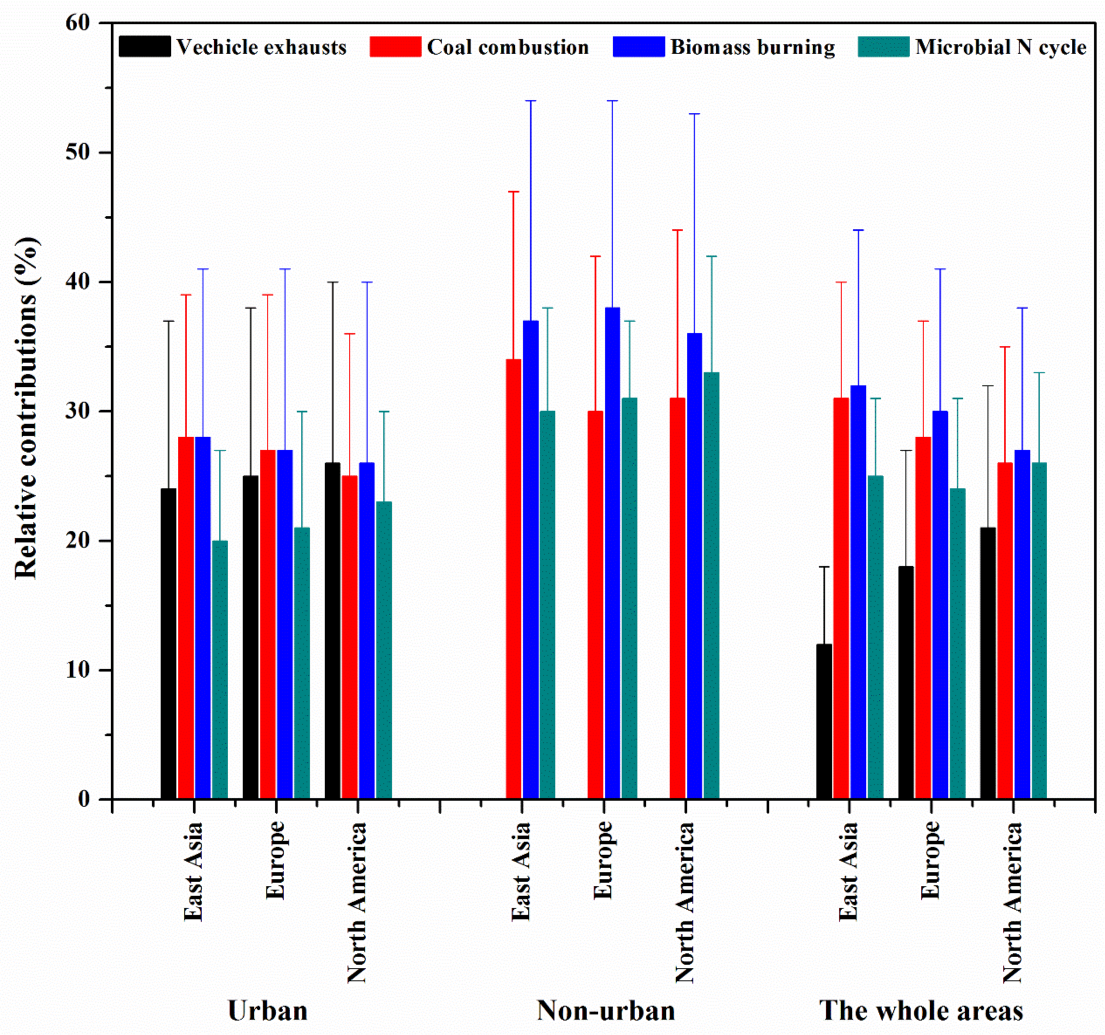

Supplementary Fig. 9 | Annual mean relative contributions of NO_x from vehicle exhausts, coal combustion, biomass burning, and microbial N cycle to precipitation NO_3^- at urban sites, non-urban sites, and the whole areas of East Asia, Europe, and North America, respectively during 2000-2017. Mean ± SD values are shown (replicates are the same as those in Supplementary Figs. 5-8). Calculations were detailed in Methods.

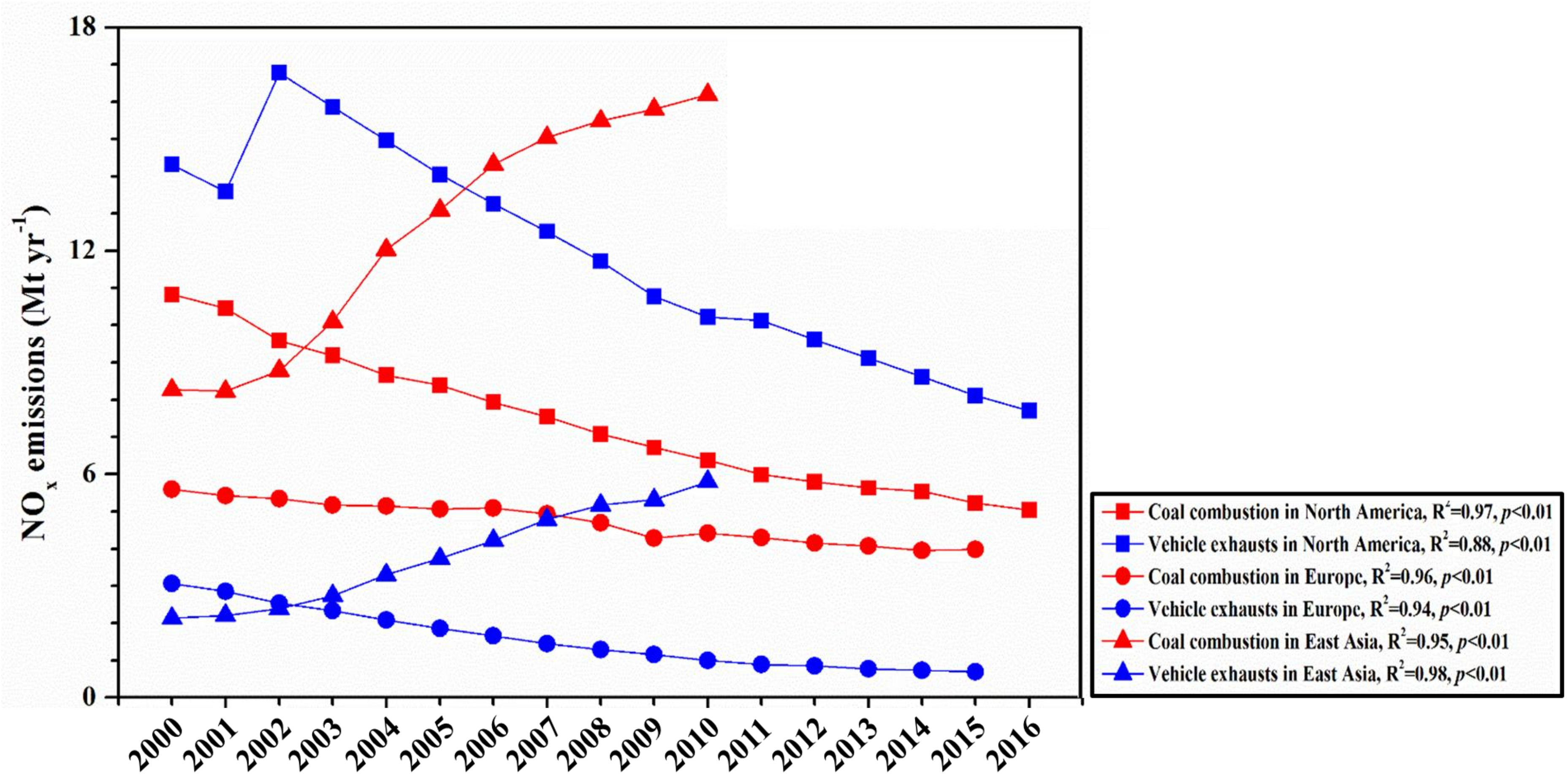

Supplementary Fig. 10 | Annual NO_x emissions from coal combustion and vehicle exhausts during 2000-2010 in East Asia, 2000-2015 in Europe, and 2000-2016 in North America. Data is cited from ref. 11 for East Asia, ref. 12 for Europe and refs. 13 and 14 for North America.

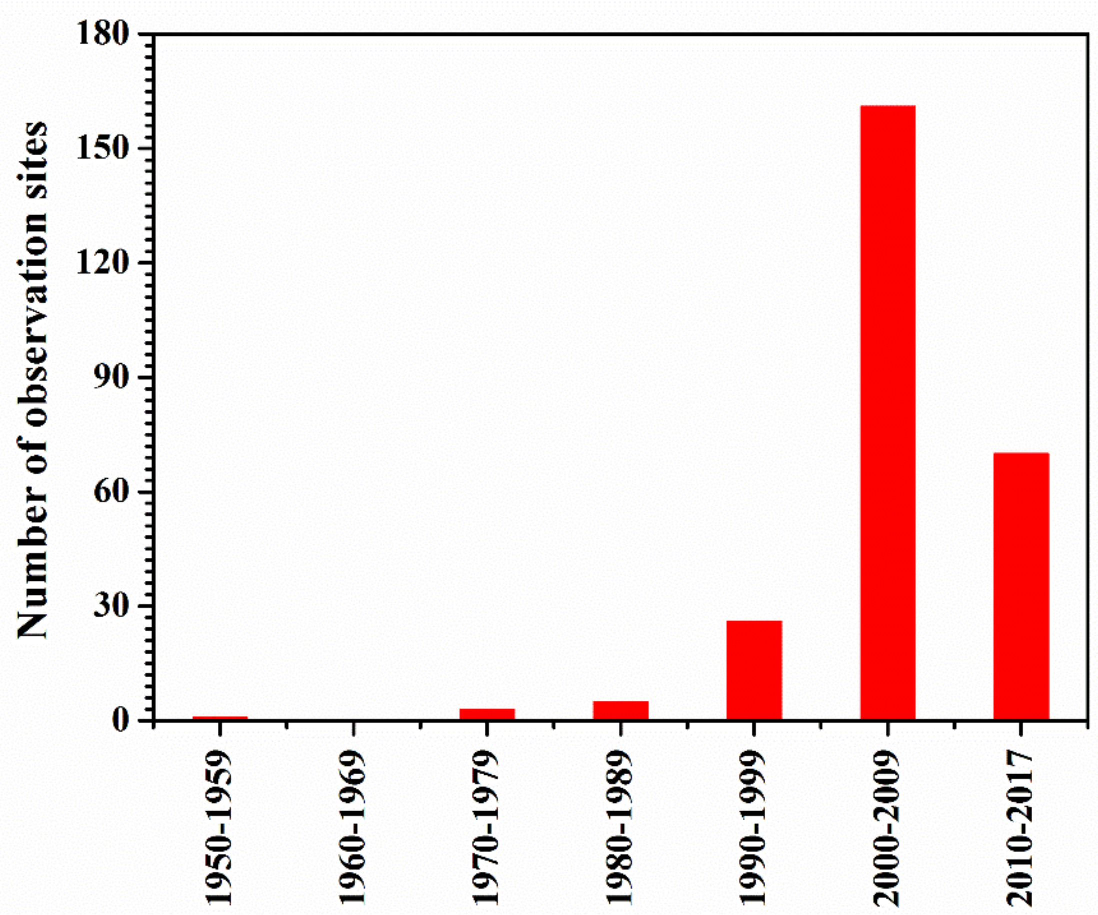

Supplementary Fig. 11 | Temporal distributions of $\delta^{15}N_{w\text{-}NO3\text{-}}$ observations from 1950 to 2017.

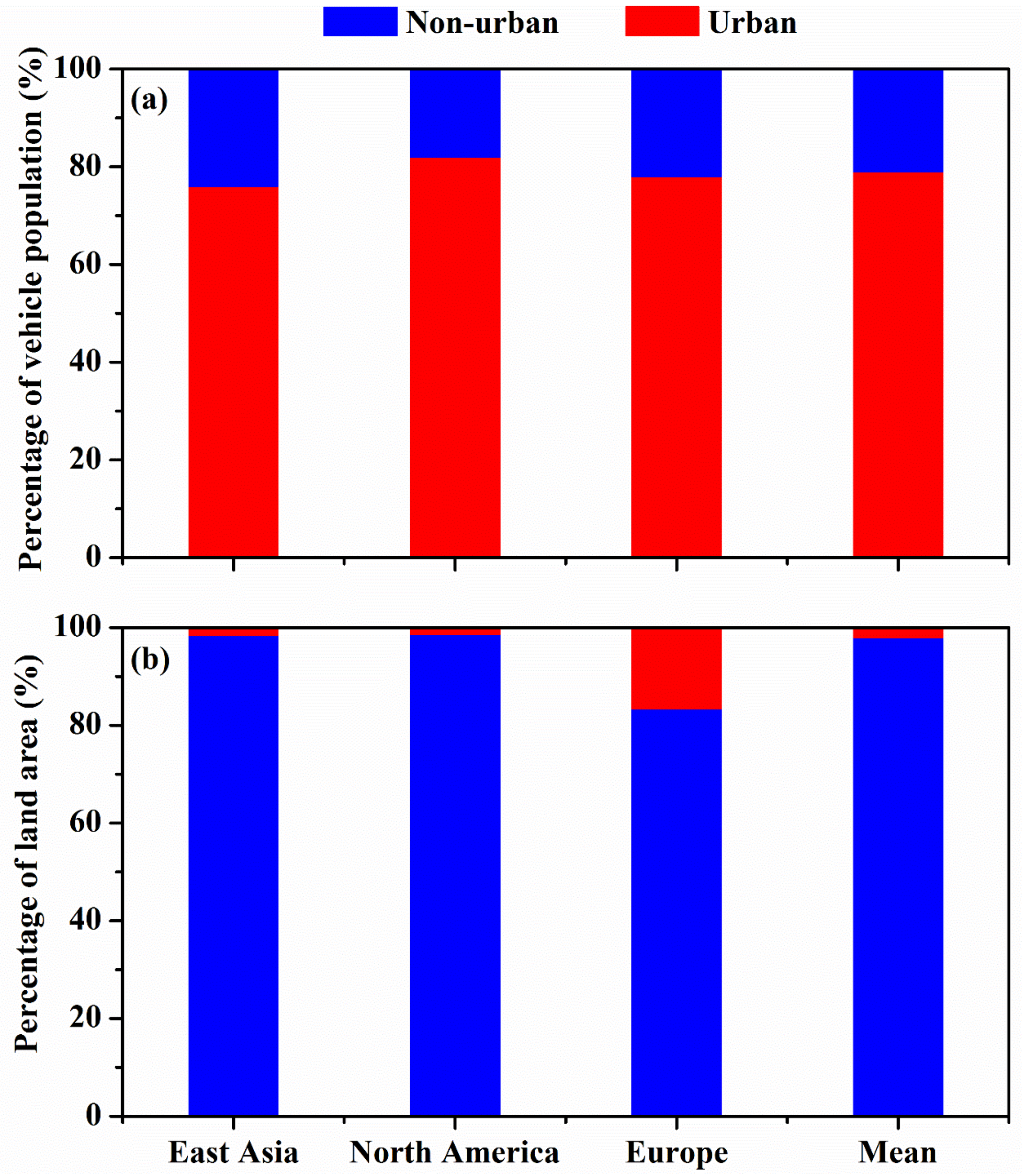

Supplementary Fig. 12 | Percentages of vehicle populations of urban and non-urban areas in the total (a), percentages of urban and non-urban areas in the total land areas in different regions (b). Calculating methods and data sources were detailed in Supplementary Tables 2 & 3.

103 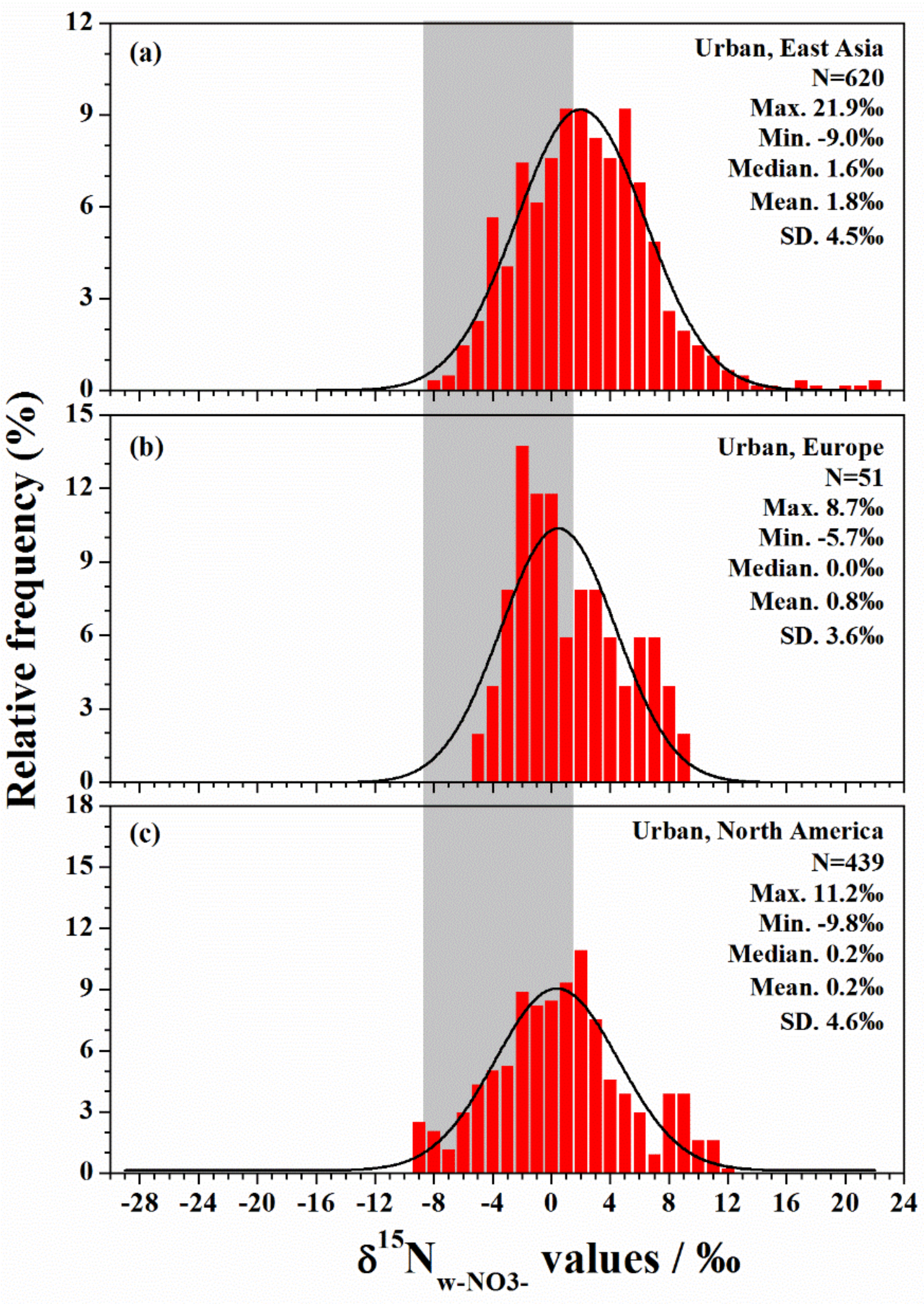
(a)
Urban, East Asia
N=620
Max. 21.9‰
Min. -9.0‰
Median. 1.6‰
Mean. 1.8‰
SD. 4.5‰
(b)
Urban, Europe
N=51
Max. 8.7‰
Min. -5.7‰
Median. 0.0‰
Mean. 0.8‰
SD. 3.6‰
(c)
Urban, North America
N=439
Max. 11.2‰
Min. -9.8‰
Median. 0.2‰
Mean. 0.2‰
SD. 4.6‰
Relative frequency (%)
δ15Nw-NO3- values / ‰

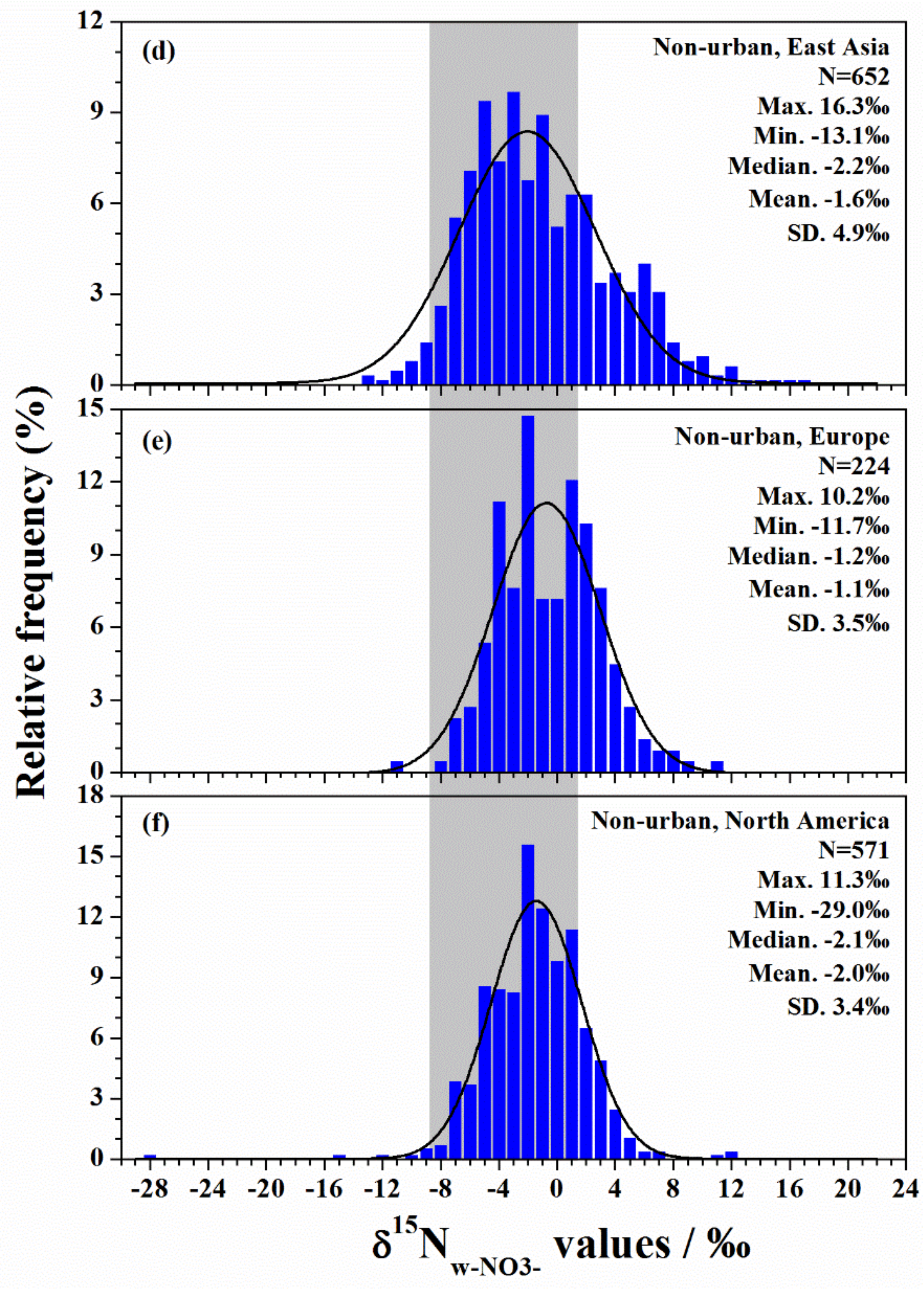

Supplementary Fig. 13 | Relative frequency histograms of $\delta^{15}N_{w\text{-}NO3\text{-}}$ values at urban (a, b, and c) and non-urban (d, e, and d) sites of East Asia, Europe, and North America, respectively. Measurements in independent observation years during 2000–2017 are shown. Trips shaded in the gray show the range of $\delta^{15}N$ of NO_x from vehicle exhausts (detailed in Supplementary Fig. 3).

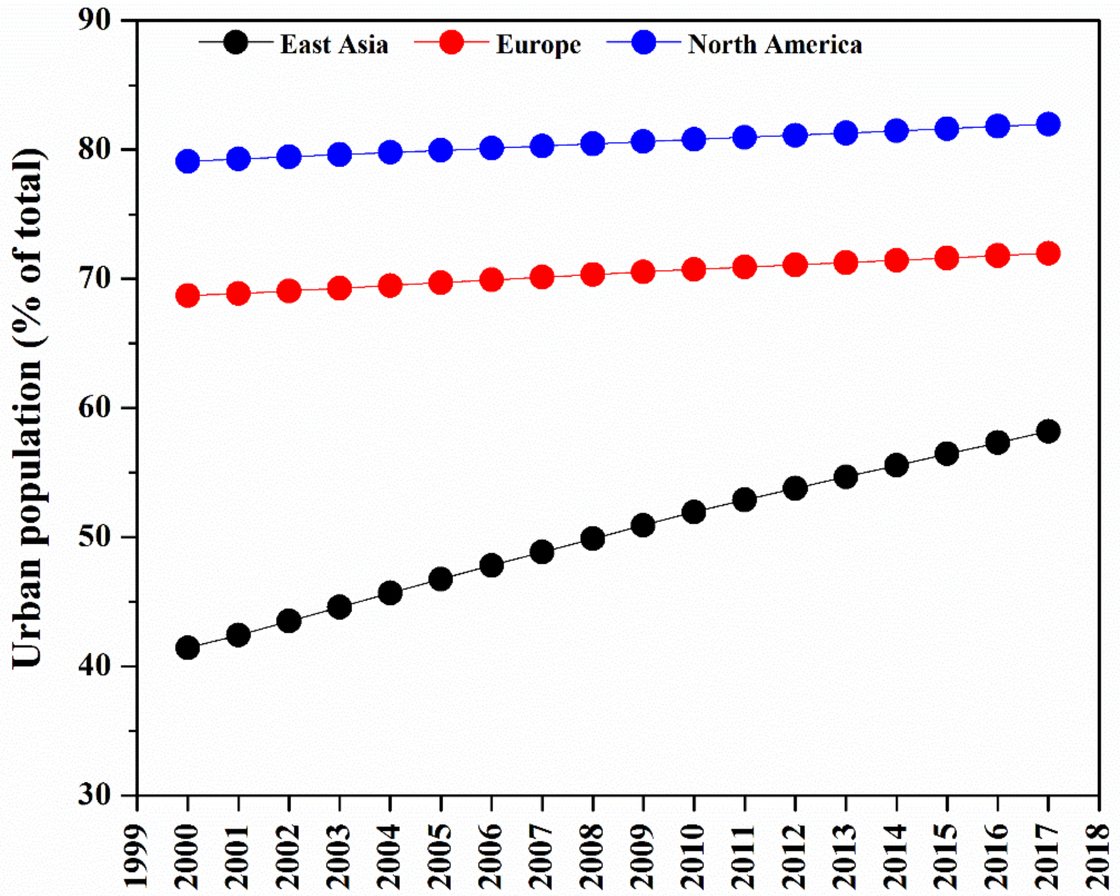

Supplementary Fig. 14 | Percentages of urban population (expressed as % of total) in East Asia, Europe, and North America between 2000–2017. Data were downloaded from ref. 15.

Supplementary Table 1 | The data sources and values of parameters used for calculating ambient $^{15}\Delta_{i\text{-}NOx\rightarrow w\text{-}NO3\text{-}}$ values. Scenario 1, mean values of regional $\delta^{15}N_{NOx}$ and f_{NO2} values, simultaneously observed values of ambient C_{NO2}, C_{HNO3}, $C_{p\text{-}NO3\text{-}}$, $\delta^{15}N_{HNO3}$, $\delta^{15}N_{p\text{-}NO3\text{-}}$, and $\delta^{15}N_{w\text{-}NO3\text{-}}$ were used. Scenario 2, non-synchronously observed values of ambient f_{NO2}, C_{NO2}, C_{HNO3}, $C_{p\text{-}NO3\text{-}}$, $\delta^{15}N_{NOx}$, $\delta^{15}N_{HNO3}$, $\delta^{15}N_{p\text{-}NO3\text{-}}$, and $\delta^{15}N_{w\text{-}NO3\text{-}}$ were used. (a) data were cited from Supplementary Texts 2 & 3; (b) data were cited from Supplementary Text 4; (c) data were cited from Supplementary Text 5; (d) data were cited from Supplementary Texts 6, 7 & 8; (e) data were cited from Supplementary Text 1.

Observations	Site, Country/Region	NO_2 (μg N m^{-3}) [a]			f_{NO2} (%) [b]			HNO_3 (μg N m^{-3}) [a]			p-NO_3^- (μg N m^{-3}) [a]		
		Mean	SD	*n*	Mean	SD	*n*	Mean	SD	*n*	Mean	SD	*n*
Scenario 1	Jülich, Germany	1.0	0.4	19	64.0	10.0	202	0.4	0.1	12	1.2	0.2	12
	Pennsylvania, USA	0.4	0.2	6	64.0	10.0	202	0.2	0.0	11	0.1	0.1	11
	Rishiri, Japan	0.3	0.1	12	64.0	10.0	202	0.1	0.0	12	0.2	0.1	12
	Sapporo, Japan	2.6	0.8	11	64.0	10.0	202	0.1	0.1	12	0.3	0.1	12
	Alberta, Canada	0.9	0.1	7	64.0	10.0	202	0.5	0.2	15	0.3	0.3	15
Scenario 2	East Asia	8.9	1.1	194	64.0	10.0	202	0.2	0.0	532	0.3	0.1	546
	Europe	1.9	0.1	200	64.0	10.0	202	0.2	0.1	200	0.5	0.3	200
	North America	7.3	1.0	162	64.0	10.0	202	0.6	0.1	420	0.2	0.1	421

(continued)

Observations	Site, Country/ Region	$\delta^{15}N_{NOx}$ (‰) [c]			$\delta^{15}N_{HNO3}$ (‰) [d]			$\delta^{15}N_{p\text{-}NO3\text{-}}$ (‰) [d]			$\delta^{15}N_{w\text{-}NO3\text{-}}$ (‰) [e]		
		Mean	SD	*n*	Mean	SD	*n*	Mean	SD	*n*	Mean	SD	*n*
Scenario 1	Jülich, Germany	-7.7	2.9	51	-2.5	0.6	12	7.3	2.6	12	-2.4	2.1	12
	Pennsylvania, USA	-7.7	2.9	51	4.3	2.8	11	7.0	3.6	11	0.0	1.7	11
	Rishiri, Japan	-7.7	2.9	51	-0.7	3.3	13	1.8	1.7	12	-3.8	2.7	13
	Sapporo, Japan	-7.7	2.9	51	2.7	1.4	13	6.2	3.1	13	-2.0	1.7	13
	Alberta, Canada	-7.7	2.9	51	-0.9	3.3	5	5.3	2.7	5	-2.1	1.6	5
Scenario 2	East Asia	-7.7	2.9	51	-1.0	4.9	49	2.3	6.4	559	-0.4	4.9	1246
	Europe	-7.7	2.9	51	-2.5	0.9	13	3.9	4.6	248	-1.7	4.1	275
	North America	-7.7	2.9	51	0.4	3.9	287	5.1	5.4	272	-1.1	4.0	994

**Supplementary Table 2 | ** Vehicles per capita, total population, urban and non-urban population (expressed as % in total), urban and non-urban vehicles, % of urban and non-urban vehicles in total, total land area, % of urban land area in total, and urban areas of different countries.

Regions	Countries	Vehicles per capita (a)	Total population ($\times10^8$) (b)	% of urban population (c)	% of non-urban population (d)	Urban vehicles ($\times10^5$)	Non-urban vehicles ($\times10^5$)	% of urban vehicles in total	% of non-urban vehicles in total	Total land areas (10^4 km^2) (e)	% of urban land area in total (f)	Urban land areas (10^4 km^2)
East Asia	China	0.06	13.71	56.0	44.0	460.7	361.9	56.0	44.0	956.3	1.0	9.5
	Japan	0.78	1.27	91.0	9.0	901.5	89.2	91.0	9.0	37.8	14.3	5.4
	Korea	0.55	0.51	82.0	18.0	230.0	50.5	82.0	18.0	10.0	16.6	1.7
North America	USA	0.82	3.21	82.0	18.0	2158.4	473.8	82.0	18.0	983.2	2.6	25.8
	Canada	0.80	0.36	81.0	19.0	231.3	54.3	81.0	19.0	998.5	0.3	2.7
Europe	England	0.71	0.65	83.0	17.0	383.6	78.6	83.0	17.0	24.4	5.9	1.5
	France	0.74	0.66	80.0	20.0	390.7	97.7	80.0	20.0	54.9	12.4	6.8
	Germany	0.77	0.82	77.0	23.0	484.4	144.7	77.0	23.0	35.7	27.5	9.8
	Italy	0.77	0.61	70.0	30.0	327.2	140.2	70.0	30.0	30.1	20.1	6.1

(a) data were cited from ref. 16.

(b-e) data were downloaded from ref. 17.

(f) data were downloaded from refs. 18–20.

Supplementary Table 3 | Urban and non-urban vehicles, % of urban and non-urban vehicles in total, total land areas, urban land areas, and % of urban and non-urban land areas of different three regions.

Regions	Urban vehicles ($\times 10^5$)	Non-urban vehicles ($\times 10^5$)	% of urban vehicles	% of non-urban vehicles	Total land areas (10^4 km^2)	Urban land areas (10^4 km^2)	% of urban land areas	% of non-urban land areas
East Asia	1592.1	501.6	76.0	24.0	1004.1	16.5	1.7	98.4
North America	2389.7	528.1	82.0	18.0	1981.6	28.5	1.4	98.6
Europe	1585.9	461.2	78.0	22.0	145.1	24.1	16.6	83.4
All three regions	5567.8	1490.8	79.0	21.0	3130.9	69.1	2.2	97.8

Supplementary References:

(1) Felix, J. D., Elliott, E. M. & Shaw, S. L. The isotopic composition of coal-fired power plant NOx: The influence of emission controls and implications for global emission inventories. Environ. Sci. Technol. 46, 3528–3535 (2012).

(2) Heaton, T. H. E. $^{15}N/^{14}N$ ratios of NO_x from vehicle engines and coal-fired power station. Tellus B. **42**, 304–307 (1990).

(3) Li, D. J. & Wang, X. M. Nitrogen isotopic signature of soil-released nitric oxide (NO) after fertilizer application. Atmos. Environ. **42**, 4747–4754 (2008).

(4) Felix, J. D., Elliott, E. M., Gish, T. J., Mcconnell, L. L. & Shaw, S. L. Characterizing the isotopic composition of atmospheric ammonia emission sources using passive samplers and a combined oxidation-bacterial denitrifier isotope ratio mass spectrometer method. Rapid. Commun. Mass. Spectrom. **27**, 2239–2246 (2013).

(5) Felix, J. D. & Elliott, E. M. The isotopic composition of passively collected nitrogen dioxide emissions: Vehicle, soil and livestock source signatures. Atmos. Environ. **92**, 359–366 (2014).

(6) Walters, W. W., Goodwin, S. R. & Michalski, G. The nitrogen stable isotope composition (^{15}N) of vehicle emitted NO_x. Environ. Sci. Technol. **49**, 2278–2285 (2015).

(7) Fibiger, D. L. & Hastings, M. G. First measurements of the nitrogen isotopic composition of NO_x from biomass burning. Environ. Sci. Technol. **50**, 11569–11574 (2016).

(8) Wang, X., Ti, C. P., Luo, Y. X. & Sun, X. L. Determination of ^{15}N natural abundance in nitrogen oxides from major anthropogenic emission sources. Acta Pedologica Sinica. **53**, 1552–1562 (2016). (in Chinese with English Abstract).

(9) Chai, J., et al. Isotopic characterization of nitrogen oxides (NO_x), nitrous acid (HONO), and nitrate (NO_3^-(p)) from laboratory biomass burning during FIREX, Atmos. Meas. Tech. **12**, 6303–6317 (2019).

(10) http://www.bp.com/en/global/corporate/energy-economics/statistical-review-of-world-energy/downloads.html

(11) Wang, S. X., Zhao, B., Cai, S. Y., Klimont, Z., Nielsen, C. P. & Morikawa, T. Emission trends and mitigation options for air pollutants in East Asia. Atmos. Chem. Phys. **14**, 6571–6603 (2014).

(12) National emission ceilings directive emissions data viewer 1990-2017. Data were downloaded from https://www.eea.europa.eu/data-and-maps/dashboards/necd-directive-data-viewer-2.

(13) Air pollutant emissions trends data. Data were downloaded from https://www.epa.gov/air-emissions-inventories/air-pollutant-emissions-trends-data.

(14) Air pollutants emissions inventory online search. Data were downloaded from https://pollution-waste.canada.ca/air-emission-inventory.

(15) https://data.worldbank.org.cn/.

(16) Dargay, J. & Gately, D. Income's effect on car and vehicle ownership, worldwide: 1960–2015. Transport. Res. A: Pol. **33**, 101−138 (1999).

(17) https://data.worldbank.org.cn/.

(18) http://www.demographia.com/db-intlualand.htm.

(19) https://new.qq.com/rain/a/20150724017824.

(20) http://finance.people.com.cn/n1/2017/1228/c1004-29734355.html.

Supplementary Text 1 | Publications of $\delta^{15}N_{w\text{-}NO3\text{-}}$ observations.

(21) Altieri, K. E., Hastings, M. G., Gobel, A. R., Peters, A. J. & Sigman, D. M. Isotopic composition of rainwater nitrate at bermuda: the influence of air mass source and chemistry in the marine boundary layer. J. Geophys. Res. Atmos. **118**, doi:10.1002/jgrd.50829 (2013).

(22) Amiri, H., Zare, M. & Widory, D. Assessing sources of nitrate contamination in the shiraz urban aquifer (Iran) using the $\delta^{15}N$ and $\delta^{18}O$ dual-isotope approach. Isot. Environ. Healt. S. **51**,392–410 (2015).

(23) Anisfeld, S. C., Barnes, R. T., Altabet, M. A. & Wu, T. X. Isotopic apportionment of atmospheric and sewage nitrogen sources in two Connecticut rivers. Environ. Sci. Technol. **41**, 6363–6369 (2007).

(24) Ansari, A. H., Hodson, A. J., Heaton, T. H. E., Kaiser, J. & Marca-Bell, A. Stable isotopic evidence for nitrification and denitrification in a high arctic glacial ecosystem. Biogeochemistry **113**, 341–357 (2012).

(25) Baranes, R. T. & Raymond, P. A. Land-use controls on sources and processing of nitrate in small watersheds: insights from dual isotopic analysis. Ecol. Appl. **20**, 1961–1978 (2010).

(26) Barnes, R. T., Raymond, P. A. & Casciotti, K. L. Dual isotope analyses indicate efficient processing of atmospheric nitrate by forested watersheds in the northeastern U.S. Biogeochemistry **90**, 15–27 (2008).

(27) Barrett, M., Hiscock, K., Pedley, S., Lerner, D., Tellam, J. & French, M. Marker species for identifying urban groundwater recharge sources: a review and case study in Nottingham, UK. Water Res. **33**, 3083–3097 (1999).

(28) Bernal, S., Hedin, L. O., Likens, G. E., Gerber, S. & Buso, D. C. Complex response of the forest nitrogen cycle to climate change. P. Nat. Acad. Sci. **109**, 3406–3411 (2012).

(29) Beyn, F., Matthias, V. & Dähnke, K. Changes in atmospheric nitrate deposition in Germany-an isotopic perspective. Environ. Pollut. **194**, 1–10 (2014).

(30) Beyn, F., Matthias, V., Aulinger, A. & Dähnke, K. Do N-isotopes in atmospheric nitrate deposition reflect air pollution levels? Atmos. Environ. **107**, 281–288 (2015).

(31) Billy, C., Billen, G., Sebilo, M., Birgand, F. & Tournebize, J. Nitrogen isotopic composition of leached nitrate and soil organic matter as an indicator of denitrification in a sloping drained agricultural plot and adjacent uncultivated riparian buffer strips. Soil Biol. Biochem. **42**, 108–117 (2010).

(32) Buda, A. R. & Dewalle, D. R. Dynamics of stream nitrate sources and flow pathways during stormflows on urban, forest and agricultural watersheds in central Pennsylvania, USA. Hydrol. Process. **23**, 3292–3305 (2010).

(33) Burns, D. A. & Kendall, C. Analysis of $\delta^{15}N$ and $\delta^{18}O$ to differentiate NO_3^- sources in runoff at two watersheds in the Catskill Mountains of New York. Water Resour. Res. doi:10.1029/2001WR000292 (2002).

(34) Buzek, F., Černý, J. & Pačes, T. The behavior of nitrogen isotopes in acidified forest soils in the Czech Republic. Water Air Soil Poll. **105**, 155−164 (1998).

(35) Campbell, D. H., Kendall, C., Chang, C. C. Y., Silva, S. R. & Tonnessen, K. A. Pathways for nitrate release from an alpine watershed: determination using $\delta^{15}N$ and $\delta^{18}O$. Water Resour. Res. doi:10.1029/2001WR000294 (2002).

(36) Campbell, J. L., Mitchell, M. J. & Mayer, B. Isotopic assessment of NO_3^- and SO_4^{2-} mobility during winter in two adjacent watersheds in the Adirondack Mountains, New York. J. Geophys. Res. Biogeo. **111**, G04007, doi:10.1029/2006JG000208 (2006).

(37) Carrillo, J. H., Hastings, M. G., Sigman, D. M. & Heubert, B. J. Atmospheric deposition of inorganic and organic nitrogen and base cations in Hawwaii. Global Biogeochem. Cy. **16,** 1076, doi:10.1029/2002GB001892 (2002).

(38) Charles, T. & Garten, J. R. Stable nitrogen isotope ratios in wet and dry nitrate deposition collected with an artificial tree. Tellus B. **48**, 60−64 (1996).

(39) Chen, Z. X., Yu, L., Liu, W. G., Lam, M. H. W., Liu, G. L. & Yin, X. B. Nitrogen and oxygen isotopic compositions of water-soluble nitrate in Taihu Lake water system, China: implication for nitrate sources and biogeochemical process. Environ. Earth. Sci. **71**, 217−223 (2014).

(40) Clow, D. W., Roop, H. A., Nanus, L., Fenn, M. E. & Sexstone, G. A. Spatial patterns of atmospheric deposition of nitrogen and sulfur using ion-exchange resin collectors in Rocky Mountain national park, USA. Atmos. Environ. **101**, 149−157 (2015).

(41) Cui, J., Zhou, F. W., Gao, M., Zhang, L. Y., Zhang, L. M., Du, K., Leng, Q. M., Zhang, Y. Z., He, D. Y., Yang, F. M. & Chan, A. A comparison of various approaches used in source apportionments for precipitation nitrogen in a mountain region of Southwest China. Environ. Pollut. **241**, 810−820 (2018).

(42) Curtis, C. J. & Heaton, T. H. E. What have stable isotope studies revealed about the nature and mechanisms of N saturation and nitrate leaching from semi-natural catchments? Ecosystems **14**, 1021−1037 (2011).

(43) Curtis, C. J., Heaton, T. H. E., Simpson, G. L., Evans, C. D., Shilland, J. & Turner, S. Dominance of biologically produced nitrate in upland waters of Great Britain indicated by stable isotopes. Biogeochemistry **111**, 535−554 (2012).

(44) Curtis, C. J., Kaiser, J., Marca, A., Anderson, N. J., Simpson, G. & Jones, V. Spatial variations in snowpack chemistry, isotopic composition of NO_3^- and nitrogen deposition from the ice sheet margin to the coast of western Greenland. Biogeosciences Discuss, **15**, 1−32 (2018).

(45) Deutsch, B., Mewes, M., Liskow, I. & Voss, M. Quantification of diffuse nitrate inputs into a small river system using stable isotopes of oxygen and nitrogen in nitrate. Org. Geochem. **37**, 1333−1342 (2006).

(46) Ding, J., Xi, B., Gao, R., He, L., Liu, H., Dai, X. & Yu, Y. Identifying diffused nitrate sources in a stream in an agricultural field using a dual isotopic approach. Sci. Total Environ. **484**, 10–18 (2014).

(47) Divers, M. T., Elliott, E. M. & Bain, D. J. Quantification of nitrate sources to an urban stream using dual nitrate isotopes. Environ. Sci. Technol. **48**, 10580–10587 (2014).

(48) Durka, W., Schulze, E. D., Gebauer, G. & Voerkeliust, S. Effects of forest decline on uptake and leaching of deposited nitrate determined from ^{15}N and ^{18}O measurements. Nature **372**, 765–767 (1994).

(49) Elliott, E. M., Kendall, C., Wankel, S. D., Burns, D. A., Boyer, E. W. & Harlin, K. Nitrogen isotopes as indicators of NO_x source contributions to atmospheric nitrate deposition across the Midwestern and Northeastern United States. Environ. Sci. Technol. **41**, 7661–7667 (2007).

(50) Emmett, B. A., Kjonaas, O. J., Gundersen, P., Koopmans, C., Tietema, A. & Sleep, D. Natural abundance of ^{15}N in forests across a nitrogen deposition gradient. Forest Ecol. Manag. **101**, 9–18 (1998).

(51) Fang, Y. T., Koba, K., Makabe, A., Takahashi, C., Zhu, W. X., Hayashi, T., Hokari, A. A., Urakawa, R., Bai, E., Houlton, B. Z., Xi, D., Zhang, S. S., Matsushita, K., Tu, Y., Liu, D. W., Zhu, F. F., Wang, Z. Y., Zhou, G. Y., Chen, D. X., Makita, T., Toda, H., Liu, X. Y., Chen, Q. S., Zhang, D. Q., Li, Y. D. & Yoh, M. Microbial denitrification dominates nitrate losses from forest ecosystems. P. Nat. Acad. Sci. **112**, 1470–1474 (2015).

(52) Fang, Y. T., Koba, K., Wang, X. M., Wen, D. Z., Li, J., Takebayashi, Y., Liu, X. Y. & Yoh, M. Anthropogenic imprints on nitrogen and oxygen isotopic composition of precipitation nitrate in a nitrogen-polluted city in southern China. Atmos. Chem. Phys. **11**, 1313–1325 (2011).

(53) Felix, J. D., Elliott, E. M., Avery, G. B., Kieber, R. J., Mead, R. N., & Willey, J. D. Isotopic composition of nitrate in sequential Hurricane Irene precipitation samples: implications for changing NO_x sources. Atmos. Environ. **106**, 191–195 (2015).

(54) Fogel, M. L. & Paerl, H. W. Isotopic tracers of nitrogen from atmospheric deposition to coastal waters. Chem. Geol. **107**, 233–236 (1993).

(55) Freyer, H. D. Seasonal trends of NH_4^+ and NO_3^- nitrogen isotope composition in rain collected at Jülich, Germany. Tellus B. **30**, 83–92 (1978).

(56) Freyer, H. D. Seasonal variation of $^{15}N/^{14}N$ ratios in atmospheric nitrate species. Tellus B. **43**, 30–44 (1991).

(57) Freyer, H. D., Kobel, K., Delmas, R. J., Kley, D. & Legrand, M. R. First results of $^{15}N/^{14}N$ ratios in nitrate from alpine and polar ice cores. Tellus B. **48**, 93–105 (1996).

(58) Fukuzaki, N. & Hayasaka, H. Seasonal variations of nitrogen isotopic ratios of ammonium and nitrate in precipitations collected in the Yahiko-Kakuda

Mountains area in Niigata Prefecture, Japan. Water Air Soil Poll. **203**, 391−397 (2009).

(59) Gao, Y. Atmospheric nitrogen deposition to Barnegat Bay. Atmos. Environ. **36**, 5783−5794 (2002).

(60) Garten, C. T. Nitrogen isotope composition of ammonium and nitrate in bulk precipitation and forest throughfall. Intern. J. Environ. Anal. Chem. **47**, 33−45 (1992).

(61) Geng, L. Alexander, B. Cole-Dai, J. Steig, E. J. Savarino, J. Sofen, E. D. & Schauer, A. J. Nitrogen isotopes in ice core nitrate linked to anthropogenic atmospheric acidity change. P. Nat. Acad. Sci. **111**, 5808−5812 (2014).

(62) Gobel, A. R., Altieri, K. E., Peters, A. J., Hastings, M. G. & Sigman, D. M. Insights into anthropogenic nitrogen deposition to the North Atlantic investigated using the isotopic composition of aerosol and rainwater nitrate. Geophys. Res. Lett. **40**, 5977−5982 (2013).

(63) Goodale, C. L., Thomas, S. A., Fredriksen, G., Elliott, E. M., Flinn, K. M., Butler, T. J. & Walter, M. T. Unusual seasonal patterns and inferred processes of nitrogen retention in forested headwaters of the Upper Susquehanna River. Biogeochemistry **93**, 197–218 (2009).

(64) Guerrieri, R., Vanguelova, E. I. Michalski, G. Heaton, T. H. E. & Mencuccini, M. Isotopic evidence for the occurrence of biological nitrification and nitrogen deposition processing in forest canopies. Global Change Biol. **21**, 4613−4626 (2015).

(65) Haberhauer, G., Gerzabek, M. H. & Krenn, A. Nitrate dynamics in an alpine forest site (mühleggerköpfl) O and N stable isotope analysis in natural water samples. Environ. Sci. Pollut. R. **9**, 37−41 (2002).

(66) Hale, R. L., Turnbull, L., Earl, S., Grimm, N., Riha, K., Michalski, G., Lohse, K. A. & Childers, D. Sources and transport of nitrogen in arid urban watersheds. Environ. Sci. Technol. **48**, 6211−6219 (2014).

(67) Hales, H. C., Ross, D. S. & Lini, A. Isotopic signature of nitrate in two contrasting watersheds of Brush Brook, Vermont, USA. Biogeochemistry **84**, 51−66 (2007).

(68) Hall, S. J., Ogata, E. M., Weintraub, S. R., Baker, M. A., Ehleringer, J. R. & Czimczik, C. I. Convergence in nitrogen deposition and cryptic isotopic variation across urban and agricultural valleys in Northern Utah. J. Geophys. Res. Biogeo. **121**, 2340−2355 (2016).

(69) Hastings, M. G., Sigman, D. M. & Lipschultz, F. Isotopic evidence for source changes of nitrate in rain at Bermuda. J. Geophys. Res. Atmos. 2003. doi:10.1029/2003JD003789 (2003).

(70) Hastings, M. G., Steig, E. J. & Sigman, D. M. Seasonal variations in N and O isotopes of nitrate in snow at Summit, Greenland: implications for the study of nitrate in snow and ice cores. J. Geophys. Res. Atmos. doi:10.1029/2004JD004991 (2004).

(71) Heaton, T. H. E. & Robertson, S. M. C. Potential canopy influences on the isotopic composition of nitrogen and sulphur in atmospheric deposition. Oecologia **109**, 600–607 (1997).

(72) Heaton, T. H. E. $^{15}N/^{14}N$ ratios of nitrate and ammonium in rain at Pretoria, south Africa. Atmo. Environ. **21**, 843–852 (1987).

(73) Heaton, T. H. E., Wynn, P. & Tye, A. M. Low $^{15}N/^{14}N$ ratios for nitrate in snow in the High Arctic (70ºN). Atmos. Environ. **38**, 5611−5621 (2004).

(74) Hernandez, D. A. S. Determining sources of nitrate in the semi-arid Rio Grande using nitrogen and oxygen isotopes. Master Thesis, The University of Tennessee. (2017).

(75) Hoering, T. The isotopic composition of the ammonia and the nitrate ion in rain. Geochim. Cosmochim. Ac. **12**, 97−102 (1957).

(76) Homyak, P. M., Sickman, J. O., Miller, A. E., Melack, J. M., Meixner, T. & Schimel, J. P. Assessing nitrogen-saturation in a seasonally dry chaparral watershed: limitations of traditional indicators of N-saturation. Ecosystems **17**, 1286−1305 (2014).

(77) Houlton, B. Z., Sigman, D. M. & Hedin, L. O. Isotopic evidence for large gaseous nitrogen losses from tropical rainforests. P. Nat. Acad. Sci. **103**, 8745−8750 (2006).

(78) Jia, G. D. & Chen, F. J. Monthly variations in nitrogen isotopes of ammonium and nitrate in wet deposition at Guangzhou, south China. Atmos. Environ. **44**, 2309−2315 (2010).

(79) Jiang, Y. J., Wu, Y. X. & Yuan, D. X. Human impacts on karst groundwater contamination deduced by coupled nitrogen with strontium isotopes in the Nandong underground river system in Yunan, China. Environ. Sci. Technol. **43**, 7676−7683 (2009).

(80) Kelley, C. J., Keller, C. K., Evans, R. D., Orr, C. H., Smith, J. L. & Harlow, B. A. Nitrate–nitrogen and oxygen isotope ratios for identification of nitrate sources and dominant nitrogen cycle processes in a tile-drained dryland agricultural field. Soil Biol. Biochem. **57**, 731−738 (2013).

(81) Knapp, A. N., Hastings, M. G., Sigman, D. M., Lipschultz, F. & Galloway, J. N. The flux and isotopic composition of reduced and total nitrogen in Bermuda rain. Mar. Chem. **120**, 83−89 (2010).

(82) Koba, K., Tokuchi, N., Wada, E., Nakajima, T. & Iwatsubo, G. Intermittent denitrification: the application of a ^{15}N natural abudance method to a forest ecosystem. Geochim. Cosmochim. Ac. **23**, 5043−5050 (1997).

(83) Kohzu, A., Imai, A., Nakajima, Y., Komatsu, K., Kawasaki, N. & Satou, T. An inexpensive auto-injection preprocessing system for N_2O by isotope ratio mass spectrometer to measure delta ^{15}N and delta ^{18}O of nitrate. Radioisotopes **60**, 231−240 (2011).

(84) Kojima, K., Sano, S., Kurisu, F. & Furumai, H. Estimation of source contribution to nitrate loading in road runoff using stable isotope analysis. Urban Water J. **14**, 337–342 (2017).

(85) Koopmans, C. J., Dam, D. V., Tietema, A. & Verstraten, J. M. Natural ^{15}N abundance in two nitrogen saturated forest ecosystems. Oecologia **111**, 470–480 (1997).

(86) Korth, F., Deutsch, B., Frey, C., Moros, C. & Voss, M. Nitrate source identification in the Baltic sea using its isotopic ratios in combination with a Bayesian isotope mixing model. Biogeosciences **11**, 4913–4924 (2014).

(87) Lee, K. S., Bong, Y. S., Lee, D., Kim, Y. & Kim, K. Tracing the sources of nitrate in the Han river watershed in Korea, using $\delta^{15}N\text{-}NO_3^-$ and $\delta^{18}O\text{-}NO_3^-$ values. Sci. Total Environ. **395**, 117–124 (2010).

(88) Leng, Q., Cui, J., Zhou, F., Du, K., Zhang, L. & Fu, C. Wet-only deposition of atmospheric inorganic nitrogen and associated isotopic characteristics in a typical mountain area, Southwestern China. Sci. Total Environ. **616**, 55–63 (2018).

(89) Li, R., Ruan, X., Bai, Y., Ma, T. & Liu, C. Effect of wheat-maize straw return on the fate of nitrate in groundwater in the Huaihe river basin, China. Sci. Total Environ. **592**, 78–85 (2017).

(90) Liang, Y., Xiao, H. Y., Liu, X. Z., Hu, Q. Q., Xie, Y, J., Cao, Y. S. & Ding, X. H. Carbon and nitrogen stable isotopes tracing nitrogen pollution in major flooding season in Lake Bang, Lake Poyang Basin. J. Lake Sci. **30**, 957–966 (2018).

(91) Liu, X. Y., Xiao, H. W., Xiao, H. Y., Song, W., Sun, X. C., & Zheng, X. D. Stable isotope analyses of precipitation nitrogen sources in Guiyang, Southwestern China. Environ. Pollut. **230**, 486–494 (2017).

(92) Lu, L., Cheng, H., Pu, X., Liu, X. & Cheng, Q. Nitrate behaviors and source apportionment in an aquatic system from a watershed with intensive agricultural activities. Environ. Sci. Proc. Imp. **17**, 131–144 (2015).

(93) Mara, P., Mihalopoulos, N., Gogou, A., Daehnke, K., Schlarbaum, T. & Emeis, K. C. Isotopic composition of nitrate in wet and dry atmospheric deposition on Crete in the eastern Mediterranean Sea. Global Biogeochem. Cy. **23**, GB4002, doi:10.1029/2008GB003395 (2009).

(94) Moore, H. Isotopic measurement of atmospheric nitrogen compounds. Tellus B. **26**, 169–174 (1974).

(95) Moore, H. The isotopic composition of ammonia, nitrogen dioxide and nitrate in the atmosphere. Atmos. Environ. **11**, 1239–1243 (1977).

(96) Morin, S., Erbland, J., Savarino, J., Domine, F., Bock, J. & Friess, U. An isotopic view on the connection between photolytic emissions of NO_x, from the Arctic snowpack and its oxidation by reactive halogens. J. Geophys. Res. Atmos. **117**(D14), doi:10.1029/2011JD016618 (2012).

(97) Morin, S., Savarino, J., Frey, M., Yan, N., Bekki, S., Bottenheim, J. W. & Martins, J. M. F. Traceing the origin and fate of NO_x in the Arctic atmosphere using stable isotopes in nitrate. Science **322**, 730–732 (2008).

(98) Mueller, C., Krieg, R., Merz, R. & Kay Knöller. Regional nitrogen dynamics in the Tereno Bode river catchment, Germany, as constrained by stable isotope patterns. Isot. Environ. Healt. S. **52**, 1–14 (2015).

(99) Mukotaka, A. A study of nitrogen oxides dynamics between urban atmosphere and the phyllosphere using triple oxygen isotope. PhD Thesis, Tokyo Institute of Technology (2014).

(100) Nanus, L., Campbell, D. H., Lehmann, C. M. B. & Mast, M. A. Spatial and temporal variation in sources of atmospheric nitrogen deposition in the Rocky Mountains using nitrogen isotopes. Atmos. Environ. **176**, 110–119 (2017).

(101) Nanus, L., Williams, M. W., Campbell, D. H., Elliott, E. M. & Kendall, C. Evaluating regional patterns in nitrate sources to watersheds in national parks of the Rocky Mountains using nitrate isotopes. Environ. Sci. Technol. **42**, 6487–6493 (2008).

(102) Nelson, D. M., Tsunogai, U. & Dong, D. Triple oxygen isotopes indicate urbanization affects sources of nitrate in wet and dry atmospheric deposition. Atmos. Chem. Phys. **18**, 6381–6392 (2018).

(103) Novak, M., Suarez, S. P., Gebauer, G., Thoma, M., Buzek, F. & Cejkova, B. Relationship between nitrogen isotope ratios of NO_3^- and N_2O in vertical porewater profiles through a polluted rain-fed peat bog. Soil Biol. Biochem. **123**, 7–9 (2018).

(104) Oelmann, Y., Kreutziger, Y., Bol, R. & Wilcke, W. Nitrate leaching in soil: tracing the NO_3^- sources with the help of stable N and O isotopes. Soil Biol. Biochem. **39**, 3024–3033 (2007).

(105) Osaka, K., Ohte, N., Koba, K., Yoshimizu, C., Katsuyama, M., Tani, M., Tayasu, I. & Nagata, T. Hydrological influences on spatiotemporal variations of ^{15}N and ^{18}O of nitrate in a forested headwater catchment in central Japan: denitrification plays a critical role in ground water. J. Geophys. Res. Biogeo. **115**, G02021, doi:10.1029/2009JG000977 (2010).

(106) Panno, S. V., Kelly, W. R., Hackley, K. C., Hwang, H. H. & Martinsek, A. T. Sources and fate of nitrate in the Illinois river basin, Illinois. J. Hydrol. **359**, 174–188 (2008).

(107) Pardo, L. H., Kendall, C., Pett-Ridge, J. & Chang, C. C. Y. Evaluating the source of streamwater nitrate using $\delta^{15}N$ and $\delta^{18}O$ in nitrate in two watersheds in New Hampshire, USA. Hydrol. Process. **18**, 2699–2712 (2004).

(108) Piatek, K. B., Mitchell, M. J., Silva, S. R. & Kendall, C. Sources of nitrate in snowmelt discharge: evidence from water chemistry and stable isotopes of nitrate. Water Air Soil Poll. **165**, 13–35 (2005).

(109) Pichlmayer, F., Schoner, W., Seibert, P., Stichler, W. & Wagenbach, D. Stable isotope analysis for characterization of pollutants at high elevation alpine sites. Atmos. Environ. **32**, 4075–4085 (1998).

(110) Popescu, R., Mimmo, T., Dinca, O. R., Capici, C., Costinel, D. & Sandru, C. Using stable isotopes in tracing contaminant sources in an industrial area: a case study on the hydrological basin of the Olt river, Romania. Sci. Total Environ. **533**, 17–23 (2015).

(111) Riha, K. M. The use of stable isotopes to constrain the nitrogen cycle. PhD Thesis, Purdue University. (2014).

(112) Rose, L. A., Elliott, E. M. & Adams, M. B. Triple nitrate isotopes indicate differing nitrate source contributions to streams across a nitrogen saturation gradient. Ecosystems, **18**, 1209–1223 (2015).

(113) Russell, K. M., Galloway, J. N., Macko, S. A., Moody, J. L. & Scudlark, J. R. Sources of nitrogen in wet deposition to the Chesapeake Bay Region. Atmos. Environ. **32**, 3923–3927 (1998).

(114) Savard, M. M., Cole, A., Smirnoff, A. & Vet, R. $\delta^{15}N$ values of atmospheric N species simultaneously collected using sector-based samplers distant from sources-isotopic inheritance and fractionation. Atmos. Environ. **162**, 11–22 (2017).

(115) Savarino, J., Kaiser, J., Morin, S., Sigman, D. M. & Thiemens, M. H. Nitrogen and oxygen isotopic constraints on the origin of atmospheric nitrate in coastal Antarctica. Atmos. Chem. Phys. **7**, 1925–1945 (2007).

(116) Schiff, S. L., Devito, K. J., Elgood, R. J., McCrindle, P. M. & Spoelstra, J. Two adjacent forested catchments: dramatically different NO_3^- export. Water Resour. Res. **38,** 1292, doi:10.1029/2000WR000170 (2002).

(117) Schwarz, M. T. & Wilcke, O. W. Stable N isotope composition of nitrate reflects n transformations during the passage of water through a montane rain forest in Ecuador. Biogeochemistry **102**, 195–208 (2011).

(118) Sebestyen, S. D., Boyer, E. W., Shanley, J. B., Kendall, C., Doctor, D. H., Aiken, G. R. & Ohte, N. Sources, transformations, and hydrological processes that control stream nitrate and dissolved organic matter concentrations during snowmelt in an upland forest. Water Resour. Res. **44**, 12, doi:10.1029/2008WR006983 (2008).

(119) Shi, J., Ohte, N., Tokuchi, N., Imamura, N., Nagayama, M. & Oda, T. Nitrate isotopic composition reveals nitrogen deposition and transformation dynamics along the canopy-soil continuum of a suburban forest in Japan. Rapid Commun. Mass Sp. **28**, 2539–2549 (2014).

(120) Smirnoff, A., Savard, M. M., Vet, R. & Simard, M. C. Nitrogen and triple oxygen isotopes in near-road air samples using chemical conversion and thermal decomposition. Rapid Commun. Mass Sp. **26**, 2791–2804 (2012).

(121) Spoelstra, J., Schiff, S. L., Elgood, R. J., Semkin, R. G., & Jeffries, D. S. Tracing the sources of exported nitrate in the Turkey Lakes watershed using $^{15}N/^{14}N$ and $^{18}O/^{16}O$ isotopic ratios. Ecosystems **4**, 536–544 (2001).

(122) Spoelstra, J., Schiff, S. L., Jeffries, D. & Semkin, R. Effect of storage on the isotopic composition of nitrate in bulk precipitation. Environ. Sci. Technol. **38**, 4723–4727 (2004).

(123) Sun L. Y. Atmospheric nitrogen and phosphorus deposition in Nanjing and effects of simulated nitrogen deposition on gaseous emissions of soils. PhD Thesis, Nanjing Agricultural University. (2014). (in Chinese with English abstract)

(124) Templer, P. H., Weathers, K. C., Ewing, H. A., Dawson, T. E., Mambelli, S., Lindsey, A. M., Webb, J., Boukili, V. K. & Firestone, M. K. Fog as a source of nitrogen for redwood trees: evidence from fluxes and stable isotopes. J. Ecol. **103**, 1397–1407 (2015).

(125) Templer, P. H., Weathers, K. C., Lindsey, A., Lenoir, K. & Scott, L. Atmospheric inputs and nitrogen saturation status in and adjacent to class I wilderness areas of the Northeastern US. Oecologia **177**, 5–15 (2015).

(126) Ti, C., Wang, X. & Yan, X. Determining $\delta^{15}N\text{-}NO_3^-$ values in soil, water, and air samples by chemical methods. Environ. Monit. Assess. **190**, 341, doi.org/10.1007/s10661-018-6712-5 (2018).

(127) Tobari, Y., Koba, K., Fukushima, K., Tokuchi, N., Ohte, N., Tateno, R., Toyoda, S., Yoshioka, T. & Yoshida, N. Contribution of atmospheric nitrate to steam-water nitrate in Japanese coniferous forests revealed by the oxygen isotope ratio of nitrate. Rapid Commun. Mass Sp. **24**, 1281–1286 (2010).

(128) Tsunogai, U., Komatsu, D. D., Daita, S., Kazemi, G. A., Nakagawa, F., Noguchi, I. & Zhang, J. Tracing the fate of atmospheric nitrate deposited onto a forest ecosystem in Eastern Asia using $\Delta^{17}O$. Atmos. Chem. Phy. **10**, 1809–1820 (2010).

(129) Tu, Y., Fang, Y., Liu, D., & Pan, Y. modifications to the azide method for nitrate isotope analysis. Rapid Commun. Mass Sp. **30**, 1213–1222 (2016).

(130) Urumu, T., Takanori, M., Takuya, O., Komatsu, D. D., Fumiko, N. & Yusuke, O. Accurate and precise quantification of atmospheric nitrate in streams, draining land of various uses by using triple oxygen isotopes as tracers. Biogeosciences **13**, 3441–3459 (2016).

(131) Wang, M., Lu, B. H., Wang, J. Q., Zhang, H, W., Guo, L. & Lin, H. Using dual isotopes and a bayesian isotope mixing model to evaluate nitrate sources of surface water in a drinking water source watershed, East China. Water, **8**, 355, doi:10.3390/w8080355 (2016).

(132) Wankel, S. D., Chen, Y., Kendall, C., Post, A. F. & Paytan, A. Sources of aerosol nitrate to the Gulf of Aqaba: evidence from $\delta^{15}N$ and $\delta^{18}O$ of nitrate and trace metal chemistry. Mar. Chem. **120**, 90–99 (2010).

(133) Wasiuta, V., Lafrenière, M. J., Norman, A. L. & Hastings, M. Summer deposition of sulfate and reactive nitrogen to two alpine valleys in the Canadian Rocky Mountains. Atmos. Environ. **101**, 270–285 (2015).

(134) Watanabe, K., Kohzu, A., Suda, W., Yamamura, S., Takamatsu, T. & Takenaka, A. Microbial nitrification in throughfall of a Japanese cedar associated with archaea from the tree canopy. SpringerPlus, **5**, 1596, doi:10.1186/s40064-016-3286-y (2016).

(135) Wexler, S. K., Goodale, C. L., McGuire, K. J., Bailey, S. W. & Groffman, P. M. Isotopic signals of summer denitrification in a northern hardwood forested catchment. P. Nat. Acad. Sci. **111**, 16413−16418 (2014).

(136) Wu, J. H., Nishijima, Y. & Satake, H. Seasonal variation of $\delta^{15}N$ of nitrate ion found in precipitation at Toyama. Geochemistry **42**, 1−11 (2008). (in Japanese with English abstract)

(137) Xia, Y., Li, Y., Zhang, X. & Yan, X. Nitrate source apportionment using a combined dual isotope, chemical and bacterial property, and Bayesian model approach in river systems. J. Geophys. Res. Biogeo. **122**, 2–14, doi:10.1002/2016JG003447 (2017).

(138) Xiao, H. Y. & Liu, C. Q. Sources of nitrogen and sulfur in wet deposition at Guiyang, southwest China. Atmos. Environ. **26**, 5121−5130 (2002).

(139) Xing, M., Liu, W., Wang, Z., & Hu, J. Relationship of nitrate isotopic character to population density in the loess plateau of Northwest China. Appl. Geochem. **35**, 110−119 (2013).

(140) Yang, J. Y. T., Hsu, S. C., Dai, M. H., Hsiao, S. S. Y. & Kao, S. J. Isotopic composition of water-soluble nitrate in bulk atmospheric deposition at Dongsha Island: sources and implications of external N supply to the northern South China Sea. Biogeosciences **11**, 1833−1846 (2014).

(141) Yeon, J., Gautam, M. K., Kim, I., Lee, S., Lee, D. & An, H. J. Isotopic composition of throughfall nitrates in suburban forests with different vegetations. Geosci. J. **19**, 167–175 (2015).

(142) Yu, L. F., Li, B. W., Zhang, Y. & Wang, Z. W. Natural abundance of nitrogen isotopes for ammonium nitrogen in rainfall and throughfall at some sites in China. Environ. Chem. **32**, 1599–1603 (2013).

(143) Yu, Q., Wang, F., Li, X., Yan, W., Li, Y. & Lv, S. Tracking nitrate sources in the Chaohu lake, China, using the nitrogen and oxygen isotopic approach. Environ. Sci. Pollut. Res. **25**, 19518–19529 (2018).

(144) Yue, F. J., Li, S. L., Liu, C. Q., Lang, Y. C. & Ding, H. Sources and transport of nitrate constrained by the isotopic technique in a karst catchment: an example from Southwest China. Hydrol. Process. **29**, 1883–1893 (2015).

(145) Yue, F. J., Liu, C. Q., Li, S. L., Zhao, Z. Q., Liu, X. L., Ding, H., Liu, B. J. & Zhong, J. Analysis of $\delta^{15}N$ and $\delta^{18}O$ to identify nitrate sources and transformations in Songhua River, Northeast China. J. Hydrol. **519**, 329–339 (2014).

(146) Zhang, Y., Liu, X. J, Fangmeier, A., Goulding, K. T. W. & Zhang, F. S. Nitrogen inputs and isotopes in precipitation in the North China Plain. Atmos. Environ. **42**, 1436–1448 (2008).

(147) Zhang, Y., Shi, P., Li, F. D., Wei, A. L., Song, J. X. & Ma, J. J. Quantification of nitrate sources and fates in rivers in an irrigated agricultural area using environmental isotopes and a Bayesian isotope mixing model. Chemosphere. **208**, 493–501 (2018).

(148) Zheng, M., Sheng, Y., Sun, R., Tian, C. & Mortimer, R. J. G. Identification and quantification of nitrogen in a reservoir, Jiaodong Peninsula, China. Water Environ. Res. **89**, 369–377 (2017).

Supplementary Text 2 | Publications of simultaneous observations of HNO_3, p-NO_3^-, and NO_2 concentrations in the atmosphere.

(149) Elliott, E., Kendall, C., Boyer, E. W., Burns, D. A., Lear, G., Golden, H., Harlin, K., Bytnerowicz, A., Butler, T. & Glatz, R. Dual nitrate isotopes in dry deposition: Utility for partitioning NO_x source contributions to landscape nitrogen deposition. J. Geophy. Res. Biogeo. **114**, G04020, doi: 10.1029/2008JG000889 (2009).

(150) Freyer, H. Seasonal variation of $^{15}N/^{14}N$ ratios in atmospheric nitrate species. Tellus B. **43**, 30–44 (1991).

(151) Freyer, H., Kley, D., Volz Thomas, A. & Kobel, K. On the interaction of isotopic exchange processes with photochemical reactions in atmospheric oxides of nitrogen. J. Geophy. Res. Atmos. **98**, 14791–14796 (1993).

(152) Nelson, D. M., Tsunogai, U., Ding, D., Ohyama, T., Komatsu, D. D., Nakagawa, F., Noguchi, I. & Yamaguchi, T. Triple oxygen isotopes indicate urbanization affects sources of nitrate in wet and dry atmospheric deposition. Atmos. Chem. Phy. **18**, 6381–6392 (2018).

(153) Savard, M. M., Cole, A., Smirnoff, A. & Vet, R. $\delta^{15}N$ values of atmospheric N species simultaneously collected using sector-based samplers distant from sources-isotopic inheritance and fractionation. Atmos. Environ. **162**, 11–22 (2017).

(154) Wojtal, P. K., Miller, D. J., O'Connor, M., Clark, S. C. & Hastings, M. G. Automated, high-resolution mobile collection system for the nitrogen isotopic analysis of NO_x. J. Vis. Exp. **118**, e54962, doi:10.3791/54962 (2016).

Supplementary Text 3 │ Data sources of observations of HNO_3, p-NO_3^-, and NO_2 concentrations in the ambient atmosphere.

(155) https://emep.int/publ/common_publications.html#2019

(156) https://monitoring.eanet.asia/document/public/index

(157) https://www3.epa.gov/ttn/amtic/supersites.html

Supplementary Text 4 | Publications of observations of ambient $f_{NO2/NOx}$ values.

(158) Cyrys, J., et al. Variation of NO_2 and NO_x concentrations between and within 36 European study areas: results from the escape study. Atmos. Environ. **62**, 374–390 (2012).

(159) Grundstrom, M., Hak, C., Chen, D., Hallquist, M. & Pleijel, H. Variation and co-variation of PM_{10}, particle number concentration, NO_x and NO_2 in the urban air-relationships with wind speed, vertical temperature gradient and weather type. Atmos. Environ. **120**, 317–327 (2015).

(160) https://monitoring.eanet.asia/document/public/index

(161) Matsui, H., Koike, M., Takegawa, N., Kondo, Y., Griffin, R. J., Miyazaki, Y., Yokouchi, Y. & Ohara, T. Secondary organic aerosol formation in urban air: Temporal variations and possible contributions from unidentified hydrocarbons. J. Geophys. Res. Atmos. **114**, D04201, doi:10.1029/2008JD010164 (2009).

(162) Mavroidis, I. & Chaloulakou, A. Long-term trends of primary and secondary NO_2 production in the Athens area. variation of the NO_2/NO_x ratio. Atmos. Environ. **45**, 6872–6879 (2011).

(163) Melkonyan, A. & Kuttler, W. Long-term analysis of NO, NO_2 and O_3 concentrations in North Rhine-Westphalia, Germany. Atmos. Environ. **60**, 316–326 (2012).

(164) Rao, M. & George, L. A. Using the NO_2/NO_x Ratio to understand the spatial heterogeneity of secondary pollutant formation capacity in urban atmospheres. Agu Fall Meeting Abstracts. A33F–3265, (2014).

(165) Richmond-Bryant, J., Owen, R. C., Graham, S., Snyder, M., McDow, S., Oakes, M. & Kimbrough, S. Estimation of on-road NO_2 concentrations, NO_2/NO_x ratios, and related roadway gradients from near-road monitoring data. Air Qual. Atmos. Heal. **10**, 611–625 (2017).

Supplementary Text 5 | Publications of ambient $\delta^{15}N_{NOx}$ observations.

(166) Dahal, B. & Hastings, M. G. Technical considerations for the use of passive samplers to quantify the isotopic composition of NO_x and NO_2 using the denitrifier method. Atmos. Environ. **143**, 60–66 (2016).

(167) Fibiger, D. L., Hastings, M. G., Lew, A. F., Peltier, R. E. Collection of NO and NO_2 for isotopic analysis of NO_x emissions. Anal. Chem. **86**, 12115–12121 (2014).

(168) Savard, M. M., Cole, A., Smirnoff, A., Vet, R. $\delta^{15}N$ values of atmospheric N species simultaneously collected using sector-based samplers distant from sources-isotopic inheritance and fractionation. Atmos. Environ. **162**, 11–22 (2017).

(169) Wojtal, P. K., Miller, D. J., O'Conner, M., Clark, S. C., Hastings, M. G. Automated, high-resolution mobile collection system for the nitrogen isotopic analysis of NO_x. J. Vis. Exp. **118**, e54962, doi:10.3791/54962 (2016).

Supplementary Text 6 | Publications of simultaneous $\delta^{15}N$ observations of HNO_3, p-NO_3^-, and w-NO_3^- in the atmosphere.

(170) Freyer, H. Seasonal variation of $^{15}N/^{14}N$ ratios in atmospheric nitrate species. Tellus B. **43,** 30–44 (1991).

(171) Elliott, E., Kendall, C., Boyer, E. W., Burns, D. A., Lear, G., Golden, H., Harlin, K., Bytnerowicz, A., Butler, T. & Glatz, R. Dual nitrate isotopes in dry deposition: Utility for partitioning NO_x source contributions to landscape nitrogen deposition. J. Geophy. Res. Biogeo. **114**, G04020, doi: 10.1029/2008JG00088 (2009).

(172) Nelson, D. M., Tsunogai, U., Ding, D., Ohyama, T., Komatsu, D. D., Nakagawa, F., Noguchi, I. &Yamaguchi, T. Triple oxygen isotopes indicate urbanization affects sources of nitrate in wet and dry atmospheric deposition. Atmos. Chem. Phy. **18**, 6381–6392 (2018).

(173) Savard, M. M., Cole, A., Smirnoff, A. & Vet, R. $\delta^{15}N$ values of atmospheric N species simultaneously collected using sector-based samplers distant from sources–Isotopic inheritance and fractionation. Atmos. Environ. **162**, 11–22 (2017).

Supplementary Text 7 | Publications of ambient $\delta^{15}N_{HNO3}$ observations.

(174) Bell, M. D. Using stable isotopes of nitrogen and oxygen as environmental indicators of nitrogen deposition in the Sonoran Desert. PhD Thesis, University of California. (2012).

(175) Elliott, E. M., Kendall, C., Boyer, E. W., Burns, D. A., Lear, G. G., Golden, H. E., Harlin, K., Bytnerowicz, A. T., Butler, J. & Glatz, R. Dual nitrate isotopes in dry deposition: Utility for partitioning NO_x source contributions to landscape nitrogen deposition, J. Geophys. Res. Atmos. **114**, G04020, doi:10.1029/2008JG000889 (2009).

(176) Felix, J. D. & Elliott, E. M. Isotopic composition of passively collected nitrogen dioxide emissions: vehicle, soil and livestock source signatures. Atmos. Environ. **92**, 359–366 (2014).

(177) Freyer, H. D. Seasonal variation of $^{15}N/^{14}N$ ratios in atmospheric nitrate species. Tellus B. **43**, 30–40 (1991).

(178) Kawashima, H. Formation mechanism and source apportionment of ammonium and nitrate ions in aerosol using nitrogen isotopes. J. Aerosol Res. **29**, 110–116 (2014). (in Japanese with English abstract)

(179) Nelson, D. M., Tsunogai, U. & Dong, D. Triple oxygen isotopes indicate urbanization affects sources of nitrate in wet and dry atmospheric deposition. Atmos. Chem. Phys. **18**, 6381–6392 (2018).

(180) Redling, K., Elliott, E., Bain, D. & Sherwell, J. Highway contributions to reactive nitrogen deposition: tracing the fate of vehicular NO_x using stable isotopes and plant biomonitors. Biogeochemistry **116**, 261–274 (2013).

(181) Savard, M. M., Cole, A., Smirnoff, A. & Vet, R. $\delta^{15}N$ values of atmospheric N species simultaneously collected using sector-based samplers distant from sources- isotopic inheritance and fractionation. Atmos. Environ. **162**, 11–22 (2017).

(182) Ti, C. P., Wang, X. & Yan, X. Y. Determining $\delta^{15}N\text{-}NO_3^-$ values in soil, water, and air samples by chemical methods. Environ. Monit. Assess. **190**, 341, doi.org/10.1007/s10661-018-6712-5 (2018).

Supplementary Text 8 | Publications of $\delta^{15}N_{p\text{-}NO3\text{-}}$ observations.

(183) Baker, A. R., Weston, K., Kelly, S. D., Voss, M., Streu, P. & Cape, J. N. Dry and wet deposition of nutrients from the tropical Atlantic atmosphere: Links to primary productivity and nitrogen fixation. Deep-Sea Res. **54**, 1704–1720 (2007).

(184) Bell, M. D. Using stable isotopes of nitrogen and oxygen as environmental indicators of nitrogen deposition in the Sonoran Desert. PhD Thesis, University of California. (2012).

(185) Beyn, F., Matthias, V., Aulinger, A. & DaHnke, K. Do N-isotopes in atmospheric nitrate deposition reflect air pollution levels? Atmos. Environ. **107**, 281–288 (2015).

(186) Beyn, F., Matthias, V. & Dahnke, K. Changes in atmospheric nitrate deposition in Germany-An isotopic perspective. Environ. Pollt. **194**, 1–10 (2014).

(187) Chang, Y., Zhang, Y., Tian, C., Zhang, S., Ma, X., Cao, F., Liu, X., Zhang, W., Kuhn, T. & Lehmann, M. F. Nitrogen isotope fractionation during gas-to-particle conversion of NO_x to NO_3^- in the atmosphere-implications for isotope-based NO_x source apportionment, Atmos. Chem. Phys. **18**, 11647–11661 (2018).

(188) Charles, T. & Garten, J. R. Stable nitrogen isotope ratios in wet and dry nitrate deposition collected with an artificial tree. Tellus B. **48**, 60–64 (1996).

(189) Gobel, A. R., Altieri, K. E., Peters, A. J., Hastings, M. G. & Sigman, D. M. Insights into anthropogenic nitrogen deposition to the North Atlantic investigated using the isotopic composition of aerosol and rainwater nitrate. Geophys. Res. Lett. **40**, 5977–5982 (2013).

(190) Guha, T., Lin, C. T., Bhattacharya, S. K., Mahajan, A. S., Ou-Yang, C. F., Lan, Y. P., Hsu, S. C. & Liang, M. C. Isotopic ratios of nitrate in aerosol samples from Mt. Lulin, a high-altitude station in central Taiwan. Atmos. Environ. **154,** 53–69 (2017).

(191) Hall, S. J., Ogata, E. M., Weintraub, S. R., Baker, M.A., Ehleringer, J. R., Czimczik, C. I. & Bowling, D. R. Convergence in nitrogen deposition and cryptic isotopic variation across urban and agricultural valleys in northern Utah, J. Geophys. Res. Biogeo. **121**, 2340–2355 (2016).

(192) He, P. Z., Xie, Z. Q., Chi, X. Y., Yu, X. W., Fan, S. D., Kang, H., Liu, C. & Zhan, H. C. Atmospheric $\Delta^{17}O(NO_3^-)$ reveals nocturnal chemistry dominates nitrate production in Beijing haze. Atmos. Chem. Phys. **18**, 14465–14476 (2018).

(193) Heaton, T. H. E. $^{15}N/^{14}N$ ratios of nitrate and ammonium in rain at Pretoria, South Africa. Atmos. Environ. **21**, 843–852 (1987).

(194) Kawashima, H. & Kurahashi, T. Inorganic ion and nitrogen isotopic compositions of atmospheric aerosols at Yurihonjo, Japan: implications for nitrogen sources. Atmos. Environ. **45**, 6309–6316 (2011).

(195) Kawashima, H. Formation mechanism and source apportionment of ammonium

and nitrate ions in aerosol using nitrogen isotopes. J. Aerosol Res. **29**, 110–116 (2014). (in Japanese with English abstract)

(196) King, M. Z. Evaluating NO_x sources and oxidation pathways impacting aerosol production on the Southern Ute Indian Reservation and Navajo Nation using geochemical isotopic analysis. Thesis, Purdue University. (2013)

(197) Kundu, S., Kawamura, K. & Lee, M. Seasonal variation of the concentrations of nitrogenous species and their nitrogen isotopic ratios in aerosols at Gosan, Jeju Island: implications for atmospheric processing and source changes of aerosols. J. Geophys. Res. Atmos.**115,** D20305, doi:10.1029/2009JD013323 (2010).

(198) Moore, H. The isotopic composition of ammonia, nitrogen dioxide and nitrate in the atmosphere. Atmos. Environ. **11**, 1239–1243 (1977).

(199) Morin, S., Erbland, J., Savarino, J., Domine, F., Bock, J., Friess, U., Jacobi, H.-W., Sihler, H. & Martins, J. M. F. An isotopic view on the connection between photolytic emissions of NO_x from the Arctic snowpack and its oxidation by reactive halogens, J. Geophys. Res. Atmos. **117**, D00R08, doi:10.1029/2011JD016618 (2012).

(200) Mukotaka, A. A study of nitrogen oxides dynamics between urban atmosphere and the phyllosphere using triple oxygen isotopes. PhD Thesis, Tokyo Institute of Technology. (2014)

(201) Park, Y. M., Park, K. S., Kim, H., Yu, S. M. & Kim, Y. H. Characterizing isotopic compositions of TC-C, NO_3^--N, and NH_4^+-N in $PM_{2.5}$ in South Korea: impact of China's winter heating. Environ. Pollut. **233**, 735–744 (2017).

(202) Proemse, B. C., Mayer, B., Chow, J. C. & Watson, J. G. Isotopic characterization of nitrate, ammonium and sulfate in stack $PM_{2.5}$ emissions in the Athabasca oil sands region, Alberta, Canada. Atmos. Environ. **60**, 555–563 (2012).

(203) Riha, K. M. The use of stable isotopes to constrain the nitrogen cycle. PhD Thesis, Purdue University. (2013).

(204) Savarino, J., Kaiser, J., Morin, S., Sigman, D. M. & Thiemens, M. H. Nitrogen and oxygen isotopic constraints on the origin of atmospheric nitrate in coastal Antarctica. Atmos. Chem. Phys. **7,** 1925–1945 (2007).

(205) Savarino, J., Morin, S., Erbland, J., Grannec, F., Patey, M. D., Vicars, W., Alexande, B. & Achterberg, E. P. Isotopic composition of atmospheric nitrate in a tropical marine boundary layer. P. Nat. Acad. Sci. **110**, 17668–17673 (2013).

(206) Tao, Y. L. Study on characteristics and sources of $PM_{2.5}$ pollutions in Tianjin urban area based on stable isotope technique. Master Thesis, Tianjin University. (2017). (in Chinese with English abstract)

(207) Wankel, S. D., Chen, Y., Kendall, C., Post, A. F. & Paytan, A. Sources of aerosol nitrate to the Gulf of Aqaba: evidence from $\delta^{15}N$ and $\delta^{18}O$ of nitrate and trace metal chemistry. Mar. Chem. **120**, 90–99 (2010).

(208) Xiao, H. W., Xie, L. H., Long, A. M., Ye, F., Pan, Y. P., Li, D. N., Long, Z. H.,

Chen, L., Xiao, H. Y. & Liu, C. Q. Use of isotopic compositions of nitrate in tsp to identify sources and chemistry in South China Sea. Atmos. Environ. **109**, 70–78 (2015).

(209) Yang, J. Y. T., Hsu, S. C., Dai, M. H., Hsiao, S. S.Y. & Kao, S. J. Isotopic composition of water-soluble nitrate in bulk atmospheric deposition at Dongsha Island: sources and implications of external N supply to the northern South China Sea. Biogeosciences **11**, 1833–1846 (2014).

(210) Yeatman, S. G., Spokes, L. J., Dennis, P. F. & Jickells, T. D. Can the study of nitrogen isotopic composition in size-segregated aerosol nitrate and ammonium be used to investigate atmospheric processing mechanisms? Atmos. Environ. **35**, 1337–1345 (2001).

(211) Yeatman, S. G., Spokes, L. J., Dennis, P. F. & Jickells, T. D. Comparisons of aerosol nitrogen isotopic composition at two polluted coastal sites. Atmos. Environ. **35**, 1307–1320 (2001).

(212) Zong, Z., Tan, Y., Wang, X. P., Tian, C. G., Fang, Y. T., Chen, Y. J., Fang, Y., Han, G. X., Li, J. & Zhang, G. Assessment and quantification of NO_x sources at a regional background site in North China: comparative results from a Bayesian isotopic mixing model and a positive matrix factorization model. Environ. Pollut. **242**, 1379–1386 (2018).

(213) Zong, Z., Wang, X., Tian, C., Chen, Y., Fang, Y., Zhang, F., Li, C., Sun, J. Z., Li, J. & Zhang, G. First assessment of NO_x sources at a regional background site in North China using isotopic analysis linked with modeling. Environ. Sci. Technol. **51**, 5923–5931 (2017).
