# Supplementary material for: Important contributions of non-fossil fuel nitrogen oxides emissions
Source: Nat Commun. 2021 Jan 11;12:243. doi: 10.1038/s41467-020-20356-0 (PMC7801390; doi:10.1038/s41467-020-20356-0)
Supplement: Supplementary file 2 — Source Data [file 41467_2020_20356_MOESM2_ESM.docx]

**Data for Fig. 2.**

| **East Asia** | | **Europe** | | **North America** | |
| --- | --- | --- | --- | --- | --- |
| **Urban** | **Non-urban** | **Urban** | **Non-urban** | **Urban** | **Non-urban** |
| **δ^15^N_w-NO3-_ values / ‰** | | | | | |
| 1.41 | 2.97 | 5.96 | -3.77 | 1.98 | -3.11 |
| 1.97 | -1.94 | -0.67 | -3.44 | -0.40 | 1.83 |
| 3.50 | 5.13 | -0.17 | -4.28 | -0.23 | -4.99 |
| -1.29 | 0.44 | -1.35 | -6.25 | -0.13 | -4.10 |
| -3.66 | 3.10 | 1.15 | -3.12 | -0.82 | -0.49 |
| -0.48 | -2.24 | 3.52 | 3.81 | 1.13 | -1.50 |
| 4.20 | -0.71 | -1.17 | -0.29 | -0.90 | -0.82 |
| -0.18 | -3.07 | -0.80 | -3.67 | -0.57 | -0.54 |
| 1.40 | 0.62 |  | 0.02 | -0.13 | 0.25 |
| -1.16 | 0.18 |  | 0.46 | -5.36 | -1.23 |
| 3.72 | -3.20 |  | 0.46 |  | -0.63 |
| 1.29 | -3.58 |  | 1.56 |  | -0.82 |
| 0.04 | -0.89 |  | -1.45 |  | -0.52 |
| 1.05 | -1.66 |  | -0.54 |  | -0.62 |
| -3.00 | 3.94 |  | -1.83 |  | 1.07 |
| 13.14 | 3.05 |  |  |  | -0.88 |
| 3.84 | -0.26 |  |  |  | -3.00 |
| 7.34 | 2.53 |  |  |  | -2.35 |
| 7.00 | 4.65 |  |  |  | -1.27 |
| 1.74 | 2.32 |  |  |  | -3.60 |
| -1.71 | -2.40 |  |  |  | -1.19 |
| 2.40 | -2.05 |  |  |  | -0.78 |
| -2.20 | 7.75 |  |  |  | -2.10 |
| 7.90 | 3.62 |  |  |  | 3.00 |
| -6.10 | 0.22 |  |  |  | 2.04 |
|  | 0.50 |  |  |  | 1.76 |
|  | -0.65 |  |  |  | -0.28 |
|  | -1.44 |  |  |  | 0.08 |
|  | 6.75 |  |  |  | -0.86 |
|  | 2.46 |  |  |  | -1.00 |
|  | -1.06 |  |  |  | -4.95 |
|  | -0.67 |  |  |  | -1.48 |
|  | -4.10 |  |  |  | -0.55 |
|  | -4.37 |  |  |  | -0.58 |
|  | 2.21 |  |  |  | 0.67 |
|  | -4.19 |  |  |  | -1.88 |
|  | -1.66 |  |  |  | -2.29 |
|  | -0.13 |  |  |  | -1.50 |
|  |  |  |  |  | -0.62 |
|  |  |  |  |  | -1.38 |
|  |  |  |  |  | -0.40 |
|  |  |  |  |  | -2.63 |
|  |  |  |  |  | -0.92 |
|  |  |  |  |  | -1.72 |
|  |  |  |  |  | -0.67 |
|  |  |  |  |  | -1.47 |
|  |  |  |  |  | -2.45 |
|  |  |  |  |  | -1.97 |
|  |  |  |  |  | -2.33 |
|  |  |  |  |  | -1.96 |
|  |  |  |  |  | -1.30 |
|  |  |  |  |  | -4.42 |
|  |  |  |  |  | -1.15 |
|  |  |  |  |  | 0.00 |
|  |  |  |  |  | 0.00 |
|  |  |  |  |  | -5.09 |
|  |  |  |  |  | -0.80 |
|  |  |  |  |  | 0.00 |
|  |  |  |  |  | -5.50 |
|  |  |  |  |  | -3.95 |
|  |  |  |  |  | -5.06 |
|  |  |  |  |  | -2.68 |
|  |  |  |  |  | -2.96 |
|  |  |  |  |  | -5.62 |
|  |  |  |  |  | -6.64 |
|  |  |  |  |  | -6.21 |
|  |  |  |  |  | -0.70 |
|  |  |  |  |  | -6.65 |
|  |  |  |  |  | -4.21 |
|  |  |  |  |  | -3.05 |
|  |  |  |  |  | -3.94 |
|  |  |  |  |  | -5.10 |
|  |  |  |  |  | -3.19 |

**Data for Fig. 3.**

| **East Asia** | | | | | | **Europe** | | | | | | **North America** | | | | | |
| --- | --- | --- | --- | --- | --- | --- | --- | --- | --- | --- | --- | --- | --- | --- | --- | --- | --- |
| **Urban** | | | **Non-urban** | | | **Urban** | | | **Non-urban** | | | **Urban** | | | **Non-urban** | | |
|  | **Mean** | **SD** |  | **Mean** | **SD** |  | **Mean** | **SD** |  | **Mean** | **SD** |  | **Mean** | **SD** |  | **Mean** | **SD** |
| **Year** | **δ^15^N_w-NO3-_ values / ‰** | | **Year** | **δ^15^N_w-NO3-_ values / ‰** | | **Year** | **δ^15^N_w-NO3-_ values / ‰** | | **Year** | **δ^15^N_w-NO3-_ values / ‰** | | **Year** | **δ^15^N_w-NO3-_ values / ‰** | | **Year** | **δ^15^N_w-NO3-_ values / ‰** | |
| 2006 | 1.41 |  | 2011 | 2.97 | 5.47 | 2006 | 5.96 | 1.69 | 2007 | -3.77 | 1.71 | 2006 | 1.98 | 3.75 | 2015 | -3.11 | 1.35 |
| 2006 | 1.97 | 2.41 | 2010 | -1.94 | 2.34 | 2013 | -0.17 | 4.53 | 2007 | -3.44 | 1.06 | 2011 | -0.78 | 5.49 | 2011 | 1.83 | 6.73 |
| 2009 | 3.50 | 2.57 | 2008 | 6.62 | 1.55 | 2012 | -1.35 | 2.14 | 2007 | -4.28 | 2.12 | 2006 | -0.40 | 3.19 | 2003 | -4.99 | 3.59 |
| 2001 | -1.29 | 3.55 | 2009 | 4.15 | 2.08 | 2009 | 2.10 |  | 2016 | -5.62 | 2.83 | 2006 | -0.23 | 5.16 | 2004 | -4.10 | 0.00 |
| 2009 | -3.66 | 5.51 | 2008 | 0.44 | 1.83 | 2010 | 0.96 | 0.72 | 2017 | -8.00 | 2.93 | 2000 | -0.13 | 1.93 | 2005 | 1.02 | 0.00 |
| 2016 | -0.48 | 5.97 | 2007 | 3.10 | 1.50 | 2002 | 3.52 | 1.78 | 2011 | -3.12 | 1.39 | 2000 | -0.82 | 1.50 | 2006 | -0.42 | 2.69 |
| 2010 | 4.20 | 3.62 | 2009 | -2.24 | . | 2009 | -0.30 | 0.00 | 2012 | 3.81 | 2.57 | 2013 | 3.22 | 1.22 | 2004 | -2.20 | 0.00 |
| 2011 | -0.18 | 3.38 | 2009 | -0.71 | 2.24 | 2010 | -1.60 | 0.71 | 2004 | 0.15 | 2.12 | 2014 | 0.62 | 3.25 | 2005 | -0.85 | 0.00 |
| 2010 | 1.40 | 2.40 | 2012 | -3.07 | 1.35 | 2009 | -0.80 |  | 2005 | -0.64 | 1.20 | 2015 | 3.46 | 4.64 | 2006 | -1.71 | 2.14 |
| 2005 | -1.16 | 1.91 | 2009 | 0.62 | . |  |  |  | 2011 | -3.67 | 1.05 | 2000 | -0.90 | 2.16 | 2000 | -0.82 | 1.40 |
| 2012 | 3.72 | 2.23 | 2009 | 0.18 | 1.48 |  |  |  | 2012 | 0.02 | 3.06 | 2005 | -0.57 | 2.31 | 2004 | -0.54 | 0.70 |
| 2011 | 1.29 | 2.93 | 2016 | -3.20 | 5.81 |  |  |  | 2006 | 0.46 | 2.29 | 2005 | -0.13 | 5.31 | 2010 | 0.25 | 2.50 |
| 2000 | 0.04 | 2.36 | 2016 | -3.58 | 4.98 |  |  |  | 2005 | 0.44 | 2.46 | 2010 | -5.36 | 0.74 | 2000 | -1.23 | 2.03 |
| 2010 | 1.05 | 0.05 | 2016 | -0.89 | 4.81 |  |  |  | 2006 | 0.47 | 2.34 |  |  |  | 2005 | 1.42 | 0.00 |
| 2005 | -3.00 | 2.24 | 2016 | -1.66 | 4.55 |  |  |  | 2003 | 1.56 | 1.14 |  |  |  | 2006 | -1.31 | 2.58 |
| 2006 | 13.14 | 4.70 | 2007 | 5.60 | 1.70 |  |  |  | 2005 | -0.78 | 1.13 |  |  |  | 2000 | -0.82 | 1.78 |
| 2009 | 3.84 | 8.52 | 2013 | 3.05 | 4.22 |  |  |  | 2006 | -2.02 | 1.72 |  |  |  | 2000 | -3.87 | 3.12 |
| 2012 | 7.34 | 2.37 | 2016 | -0.26 | 1.28 |  |  |  | 2012 | -0.54 | 2.39 |  |  |  | 2000 | -0.52 | 1.27 |
| 2005 | 7.00 | 5.76 | 2010 | 4.07 | 3.79 |  |  |  | 2005 | -1.68 | 2.77 |  |  |  | 2000 | -0.62 | 0.96 |
| 2005 | 1.74 | 2.96 | 2011 | -1.33 | 3.79 |  |  |  | 2006 | -1.95 | 1.79 |  |  |  | 2005 | 3.94 | 0.00 |
| 2009 | -1.71 | 3.00 | 2010 | 4.65 | 4.11 |  |  |  | 2006 | 2.90 | 2.99 |  |  |  | 2006 | 0.11 | 2.68 |
| 2005 | 2.40 | 5.36 | 2011 | 2.32 | 0.54 |  |  |  |  |  |  |  |  |  | 2005 | 0.43 | 0.00 |
| 2010 | -2.20 |  | 2011 | -2.40 | 4.68 |  |  |  |  |  |  |  |  |  | 2006 | -1.32 | 1.80 |
| 2010 | 7.90 |  | 2014 | -2.05 | 4.90 |  |  |  |  |  |  |  |  |  | 2004 | -3.00 | 0.00 |
| 2010 | -6.10 |  | 2014 | 7.75 | 0.78 |  |  |  |  |  |  |  |  |  | 2005 | -2.35 | 3.80 |
| 2010 | -2.20 | . | 2004 | 3.62 | 7.43 |  |  |  |  |  |  |  |  |  | 2005 | -1.12 | 0.00 |
| 2010 | 7.90 | . | 2005 | 0.22 | 2.38 |  |  |  |  |  |  |  |  |  | 2006 | -1.31 | 1.78 |
| 2010 | -6.10 | . | 2010 | 1.62 | 1.94 |  |  |  |  |  |  |  |  |  | 2004 | -3.60 | 0.00 |
|  |  |  | 2011 | 1.95 | 1.36 |  |  |  |  |  |  |  |  |  | 2006 | -1.19 | 2.10 |
|  |  |  | 2012 | 2.19 | 0.99 |  |  |  |  |  |  |  |  |  | 2000 | -0.78 | 1.05 |
|  |  |  | 2006 | 0.50 | . |  |  |  |  |  |  |  |  |  | 2004 | -2.10 | 0.00 |
|  |  |  | 2011 | -0.65 | 3.11 |  |  |  |  |  |  |  |  |  | 2008 | 3.00 | 3.00 |
|  |  |  | 2005 | -1.44 | 3.81 |  |  |  |  |  |  |  |  |  | 2005 | 2.04 | 0.00 |
|  |  |  | 2012 | 6.75 | 3.65 |  |  |  |  |  |  |  |  |  | 2006 | 2.04 | 3.64 |
|  |  |  | 2014 | 2.46 | 4.21 |  |  |  |  |  |  |  |  |  | 2005 | 4.44 | 0.00 |
|  |  |  | 2014 | -0.67 | 2.70 |  |  |  |  |  |  |  |  |  | 2006 | 0.87 | 2.99 |
|  |  |  | 2005 | -1.06 | 3.84 |  |  |  |  |  |  |  |  |  | 2000 | -0.28 | 2.37 |
|  |  |  | 2004 | -0.67 | 1.24 |  |  |  |  |  |  |  |  |  | 2000 | 0.08 | 2.37 |
|  |  |  | 2002 | -4.10 | 2.15 |  |  |  |  |  |  |  |  |  | 2002 | -0.86 | 3.15 |
|  |  |  | 2009 | -5.07 | 2.50 |  |  |  |  |  |  |  |  |  | 2000 | -2.98 | 8.41 |
|  |  |  | 2010 | -4.65 | 2.91 |  |  |  |  |  |  |  |  |  | 2000 | -1.48 | 2.08 |
|  |  |  | 2011 | -3.89 | 3.38 |  |  |  |  |  |  |  |  |  | 2005 | 1.84 | 0.00 |
|  |  |  | 2012 | -0.84 | 3.37 |  |  |  |  |  |  |  |  |  | 2006 | -1.35 | 3.57 |
|  |  |  | 2009 | 2.21 | 8.00 |  |  |  |  |  |  |  |  |  | 2000 | -0.58 | 1.86 |
|  |  |  | 2009 | -4.19 | 4.40 |  |  |  |  |  |  |  |  |  | 2000 | 0.67 | 2.03 |
|  |  |  | 2008 | -1.66 | 4.60 |  |  |  |  |  |  |  |  |  | 2005 | -0.21 | 0.00 |
|  |  |  | 2012 | -0.13 | 2.41 |  |  |  |  |  |  |  |  |  | 2006 | -2.43 | 2.13 |
|  |  |  |  |  |  |  |  |  |  |  |  |  |  |  | 2006 | -2.29 | 2.98 |
|  |  |  |  |  |  |  |  |  |  |  |  |  |  |  | 2000 | -1.50 | 2.23 |
|  |  |  |  |  |  |  |  |  |  |  |  |  |  |  | 2013 | 1.05 | 1.53 |
|  |  |  |  |  |  |  |  |  |  |  |  |  |  |  | 2014 | -1.87 | 3.17 |
|  |  |  |  |  |  |  |  |  |  |  |  |  |  |  | 2015 | 2.24 | 2.32 |
|  |  |  |  |  |  |  |  |  |  |  |  |  |  |  | 2000 | -0.89 | 1.62 |
|  |  |  |  |  |  |  |  |  |  |  |  |  |  |  | 2000 | -2.63 | 1.72 |
|  |  |  |  |  |  |  |  |  |  |  |  |  |  |  | 2000 | -0.92 | 2.58 |
|  |  |  |  |  |  |  |  |  |  |  |  |  |  |  | 2000 | -1.72 | 2.55 |
|  |  |  |  |  |  |  |  |  |  |  |  |  |  |  | 2006 | -0.67 | 2.16 |
|  |  |  |  |  |  |  |  |  |  |  |  |  |  |  | 2005 | -0.20 | 0.00 |
|  |  |  |  |  |  |  |  |  |  |  |  |  |  |  | 2006 | -1.89 | 4.04 |
|  |  |  |  |  |  |  |  |  |  |  |  |  |  |  | 2005 | -2.45 | 2.83 |
|  |  |  |  |  |  |  |  |  |  |  |  |  |  |  | 2000 | -1.97 | 1.74 |
|  |  |  |  |  |  |  |  |  |  |  |  |  |  |  | 2000 | -2.33 | 1.77 |
|  |  |  |  |  |  |  |  |  |  |  |  |  |  |  | 2005 | -1.96 | 1.63 |
|  |  |  |  |  |  |  |  |  |  |  |  |  |  |  | 2000 | -1.30 | 2.18 |
|  |  |  |  |  |  |  |  |  |  |  |  |  |  |  | 2000 | -4.42 | 2.70 |
|  |  |  |  |  |  |  |  |  |  |  |  |  |  |  | 2000 | 0.47 | 0.78 |
|  |  |  |  |  |  |  |  |  |  |  |  |  |  |  | 2011 | -2.37 | 3.82 |
|  |  |  |  |  |  |  |  |  |  |  |  |  |  |  | 2000 | 0.00 | 1.23 |
|  |  |  |  |  |  |  |  |  |  |  |  |  |  |  | 2005 | 0.00 | 2.50 |
|  |  |  |  |  |  |  |  |  |  |  |  |  |  |  | 2007 | -5.09 | 2.26 |
|  |  |  |  |  |  |  |  |  |  |  |  |  |  |  | 2000 | -0.80 | 2.35 |
|  |  |  |  |  |  |  |  |  |  |  |  |  |  |  | 2004 | 0.00 | 1.67 |
|  |  |  |  |  |  |  |  |  |  |  |  |  |  |  | 2004 | -5.50 | 0.00 |
|  |  |  |  |  |  |  |  |  |  |  |  |  |  |  | 2000 | -3.95 | 2.53 |
|  |  |  |  |  |  |  |  |  |  |  |  |  |  |  | 2006 | -5.06 | 1.81 |
|  |  |  |  |  |  |  |  |  |  |  |  |  |  |  | 2000 | -2.68 | 2.18 |
|  |  |  |  |  |  |  |  |  |  |  |  |  |  |  | 2000 | -2.96 | 0.90 |
|  |  |  |  |  |  |  |  |  |  |  |  |  |  |  | 2000 | -5.62 | 1.84 |
|  |  |  |  |  |  |  |  |  |  |  |  |  |  |  | 2006 | -6.64 | 1.31 |
|  |  |  |  |  |  |  |  |  |  |  |  |  |  |  | 2001 | -6.21 | 1.13 |
|  |  |  |  |  |  |  |  |  |  |  |  |  |  |  | 2007 | -0.70 | 2.74 |
|  |  |  |  |  |  |  |  |  |  |  |  |  |  |  | 2000 | -6.65 | 1.10 |
|  |  |  |  |  |  |  |  |  |  |  |  |  |  |  | 2005 | -2.75 | 0.00 |
|  |  |  |  |  |  |  |  |  |  |  |  |  |  |  | 2006 | -4.69 | 2.10 |
|  |  |  |  |  |  |  |  |  |  |  |  |  |  |  | 2010 | -3.05 | 1.91 |
|  |  |  |  |  |  |  |  |  |  |  |  |  |  |  | 2010 | -3.94 | 2.43 |
|  |  |  |  |  |  |  |  |  |  |  |  |  |  |  | 2004 | -5.10 | 0.00 |
|  |  |  |  |  |  |  |  |  |  |  |  |  |  |  | 2013 | -3.19 | 1.61 |

**Data (mean±SD) for Fig. 4.**

| Regions | Relative contributions (%) | |  | NO_x_ emissions (Mt yr^-1^) | | |
| --- | --- | --- | --- | --- | --- | --- |
|  | Fossil fuel NO_x_ | Non-fossil fuel NO_x_ | | Fossil | Non-fossil | Total |
| East Asia | 43±11 | 57±13 |  | 16.3±4.5 | 21.6±16.6 | 37.9±16.4 |
| Europe | 46±13 | 54±13 |  | 6.3±1.3 | 7.4±5.5 | 13.7±5.6 |
| North America | 47±14 | 53±13 |  | 19.3±4.6 | 21.8±18.5 | 41.1±18.8 |
